# Supplementary material for: Early stimulated immune responses predict clinical disease severity in hospitalized COVID-19 patients
Source: Commun Med (Lond). 2022 Sep 12;2:114. doi: 10.1038/s43856-022-00178-5 (PMC9466310; doi:10.1038/s43856-022-00178-5)
Supplement: Supplementary file 1 — Supplementary Information [file 43856_2022_178_MOESM1_ESM.pdf]

# Supplementary information: Early stimulated immune responses predict clinical disease severity in hospitalized COVID-19 patients

Rebecka Svanberg[1], Cameron MacPherson[2], Adrian Zucco[2], Rudi Agius[1], Tereza Faitova[1], Michael Asger Andersen[1], Caspar da Cunha-Bang[1], Lars Klingen Gjærde[1], Maria Elizabeth Engel Møller[3], Patrick Terrence Brooks[3], Birgitte Lindegaard[4,5], Adin Sejdic[4,6], Anne Ortved Gang[1,5,7], Ditte Stampe Hersby[1,7], Christian Brieghel[1,7], Susanne Dam Nielsen[5,8], Daria Podlekareva[9], Annemette Hald[8], Jakob Thaning Bay[3], Hanne Marquart[3], Jens Lundgren[2,5,8], Anne-Mette Lebech[5,8], Marie Helleberg[2,8], Carsten Utoft Niemann*[1,5] and Sisse Rye Ostrowski*[3,5]

1 Department of Hematology, Rigshospitalet, Copenhagen University Hospital, Rigshospitalet, Copenhagen, Denmark
2 PERSIMUNE Center of Excellence, Copenhagen University Hospital, Rigshospitalet, Copenhagen, Denmark
3 Department of Clinical Immunology, Copenhagen University Hospital, Rigshospitalet, Denmark
4 Department of Pulmonary and Infectious Diseases, Copenhagen University Hospital, Nordsjællands Hospital, Hillerød, Denmark
5 Department of Clinical Medicine, University of Copenhagen, Copenhagen, Denmark
6 Department of Virus & Microbiological Special Diagnostics, Division of Infectious Disease Preparedness and Research, Statens Serum Institut, Copenhagen, Denmark
7 Department of Medicine, Section of Infectious Diseases, Herlev and Gentofte Hospital, Herlev, Denmark
8 Department of Infectious Diseases, Copenhagen University Hospital, Rigshospitalet, Copenhagen, Denmark
9 Department of Respiratory Medicine, Bispebjerg Hospital, Copenhagen, Denmark

*These authors jointly supervised this work

## Supplementary Methods

### Clinical data

The definition of a pre-existing condition associated with immune suppression was established together with a team of specialist clinicians, by assessment of each patients' medical history including diagnoses, medications and previous hospital admissions. For the current cohort, pre-existing immunosuppressive conditions included active malignancy (solid or hematological) with either chemotherapy regime administered within 12 months prior or having undergone substantial surgery within 1 month prior, sero-positive rheumatoid arthritis with methotrexate-associated lung fibrosis, active human immunodeficiency virus infection/acquired immunodeficiency syndrome, chronic kidney disease ≥ grade 4, receiving immunosuppressive medication due to a solid organ transplant, or severe comorbidity with chronic insufficiency of multiple organs (severe chronic cardiac insufficiency, severe chronic obstructive pulmonary disease , and chronic kidney disease).

### Defining a clinical severity scale

Based on the clinical disease trajectories, we defined a clinical severity scale with 4 grades of disease severity (Fig 1C), modified from a previously published COVID-19 severity grading system.[24] Where the World Health Organization (WHO) COVID-19 disease severity classifications[35] are based mainly on the symptoms of the patient, the severity scale applied here was based on needed interventions. Grade 1 was defined as requiring less than 3 liters per minute (L/min) of supplemental oxygen ($O_2$) to keep peripheral blood oxygen saturation (SAT) > 92%, and would correspond mainly to WHO classification *mild-* and *moderate disease*, however may also include milder cases of WHO classification *severe disease*. Grade 2 was defined as requiring ≥3 but <6 L/min of $O_2$ to maintain SAT > 92%, and would correspond mainly to WHO classification *severe disease*. Grade 3 was defined as requiring ≥6 L/min of $O_2$ and/or being admitted to the ICU, and would correspond to more severe cases of WHO classification *severe disease* as well as cases with *critical disease* not in need of mechanical ventilation. Grade 4 was defined as being treated with mechanical ventilator support, thus corresponding to cases with WHO classification *critical disease* in need of mechanical ventilation.

### Timepoints for comparison and handling of missing samples

For five patients, samples corresponding to actual peak clinical severity were not available and, thus, these patients represented a lower grade of severity at this time point. However, since we at "Peak Severity" investigated the analyses' reflection of *ongoing disease severity*, this was insignificant. Seven patients were included in the study >7 days after initial hospitalization for different (random) reasons, and thus, baseline samples for both TruCulture and DuraClone were missing for these patients (four patients from Peak Severity Grade 1, three patients from Peak Severity Grade 3). Five additional patients did not have DuraClone samples collected in parallel with TruCulture samples at baseline, these samples were

missing for random reasons (four patients from Grade 1, and one patient from Grade 2). At “Peak Severity”, a DuraClone sample was missing at random for two patients (both from Grade 1). At “Discharge”, the two patients with death as final outcome were not included. In addition, three patients did not have TruCulture samples and seven additional patients did not have DuraClone samples due to either rapid discharge after hospital admission or transfer to another hospital.

| | Conjugate name | Isotype | Clone |
|---|---|---|---|
| FL1 | CD16-FITC | IGG1 | SZ21 |
| FL2 | CD69-PE | IGG2B | TP1.55.3 |
| FL3 | CD16-ECD | IGG1 | 3G8 |
| FL4 | CD56-PC5.5 | IGG1 | N901 |
| FL5 | CD19-PC7 | IGG1 | J3-119 |
| FL6 | CD8-APC | IGG1 | B9.11 |
| FL7 | CD4-APC-A700 | IGG1 | 13B8.2 |
| FL8 | CD3-APC-A700 | IGG1 | UCHT1 |
| FL9 | CD14-PBE | IGG2A | RMO52 |
| FL10 | CD45-KrO | IGG1 | J33 |

Supplementary Table 1. Overview over the applied 10-color flow cytometry panel (DuraClone). A special designed 10-color flowcytometry panel (DuraClone) with prefabricated dried antibodies was used. The antibodies were tested and titrated on normal blood cells by the manufacturer and saturated concentrations of antibody were added to the dry, unitized antibody panels.

| | Name | Antigen(s) |
|---|---|---|
| **1** | Leukocytes | CD45pos |
| **2** | Neutrophils | CD16pos CD45pos SShigh |
| **3** | Eosinophils | CD16neg CD45pos SShigh |
| **4** | Monocytes | CD14pos CD45pos |
| **5** | Lymphocytes | CD45pos SSlow |
| **6** | T-cells | CD3pos |
| **7** | CD4 T-cells | CD4pos CD3pos |
| **9** | CD8 T-cells | CD8pos CD3pos |
| **11** | Mature B-cells | CD45bri CD19pos |
| **12** | NK-cells | (CD16pos and/or CD56pos) CD3neg |

Supplementary Table 2. Gating strategies for the immune cell subset populations.

| Variable | p-value | Chi-squared | Bonferroni adj. p-value |
|---|---|---|---|
| NULL_IFNa | 0.406819501228911 | 2.90302649228706 | 1 |
| NULL_IFNg | 0.428426255617833 | 2.77021681562396 | 1 |
| NULL_IL10 | 0.732984853929307 | 1.28379420144126 | 1 |
| NULL_IL12 | 0.235049360491309 | 4.25666666666668 | 1 |
| NULL_IL17A | 0.264051392330532 | 3.97614678899083 | 1 |
| NULL_IL1b | 0.0492017492482389 | 7.85062955599735 | 0.44281574323415 |
| NULL_IL6 | 0.492011065546233 | 2.40873290492668 | 1 |
| NULL_IL8 | 0.276521773327879 | 3.86406455862978 | 1 |
| NULL_TNFa | 0.153711375366132 | 5.26011698797166 | 1 |
| LPS_IFNa | 0.0268211922818983 | 9.19387872796178 | 0.241390730537084 |
| LPS_IFNg | 0.0331613713076303 | 8.7262187088274 | 0.298452341768673 |
| LPS_IL10 | 0.0517886778521297 | 7.73626482213439 | 0.466098100669168 |
| LPS_IL12 | 0.0146877883071953 | 10.5108036890646 | 0.132190094764758 |
| LPS_IL17A | 0.0309981514289856 | 8.87511969621925 | 0.278983362860871 |
| LPS_IL1b | 0.00565167164757089 | 12.5750988142292 | 0.050865044828138 |
| LPS_IL6 | 0.0111705582083377 | 11.1051712779974 | 0.100535023875039 |
| LPS_IL8 | 0.136123082611007 | 5.54242424242425 | 1 |
| LPS_TNFa | 0.00467436646993576 | 12.9826086956522 | 0.0420692982294219 |
| R848_IFNa | 0.0637440152845508 | 7.27099192618223 | 0.573696137560957 |
| R848_IFNg | 0.0655428650641719 | 7.20843214756258 | 0.589885785577547 |
| R848_IL10 | 0.0474686945196755 | 7.93056653491436 | 0.42721825067708 |
| R848_IL12 | 0.0424604222302661 | 8.17864325750082 | 0.382143800072395 |
| R848_IL17A | 0.0425651790097677 | 8.17316831683169 | 0.383086611087909 |
| R848_IL1b | 0.0450035680269829 | 8.04930830039527 | 0.405032112242846 |
| R848_IL6 | 0.0111258220474626 | 11.1138669301713 | 0.100132398427163 |
| R848_IL8 | 0.342179183063318 | 3.33952569169961 | 1 |
| R848_TNFa | 0.116076297669714 | 5.90991436100133 | 1 |
| cd3_IFNa | 0.619449195168037 | 1.77930283224401 | 1 |
| cd3_IFNg | 0.985873400067573 | 0.145454545454541 | 1 |
| cd3_IL10 | 0.843145301406529 | 0.826388888888892 | 1 |
| cd3_IL12 | 0.792723830426987 | 1.03524804177546 | 1 |
| cd3_IL17A | 0.910119599573251 | 0.539553532726484 | 1 |
| cd3_IL1b | 0.709841564945781 | 1.38165038002172 | 1 |
| cd3_IL6 | 0.983683280464957 | 0.160606060606057 | 1 |
| cd3_IL8 | 0.60295168971351 | 1.85541125541125 | 1 |
| cd3_TNFa | 0.98531905387061 | 0.149350649350652 | 1 |
| poly_IFNa | 0.545382556641325 | 2.13241658407665 | 1 |
| poly_IFNg | 0.270143089306768 | 3.92081067721207 | 1 |
| poly_IL10 | 0.792591067875559 | 1.03579688285571 | 1 |
| poly_IL12 | 0.355579765191473 | 3.24353070175439 | 1 |
| poly_IL17A | 0.167008932139985 | 5.06626184323859 | 1 |
| poly_IL1b | 0.0758301141372669 | 6.8797743487639 | 0.682471027235402 |
| poly_IL6 | 0.527904903441006 | 2.22058629776022 | 1 |
| poly_IL8 | 0.244972787365735 | 4.15729115175482 | 1 |
| poly_TNFa | 0.0698142148184061 | 7.06630345055309 | 0.628327933365655 |

**Supplementary Table 3. Summary statistics from Kruskal-Wallis tests comparing cytokine concentrations at baseline.** Summary statistics for Kruskal-Wallis tests comparing distribution of TruCulture cytokine concentrations between peak severity groups at baseline. Bonferroni adjustment is performed within each stimulus (9 tests).

| Variable | p-value | Chi-squared | Bonferroni adj. p-values |
|---|---|---|---|
| Neutrophils | 0.847787803629749 | 0.807017543859651 | 1 |
| Eosinophils | 0.558140345879624 | 2.06934001670843 | 1 |
| Monocytes | 0.367305558970854 | 3.16207184628237 | 1 |
| B-Cells | 0.679958491524767 | 1.51002506265664 | 1 |
| T-Cells | 0.36245553184943 | 3.19548872180452 | 1 |
| CD4+ T-Cells | 0.821252675236512 | 0.917293233082709 | 1 |
| CD8+ T-Cells | 0.253546518703038 | 4.07435254803675 | 1 |
| NK-Cells | 0.309037588117567 | 3.59189640768589 | 1 |

**Supplementary Table 4. Summary statistics from Kruskal-Wallis tests comparing immune cell subset counts at baseline.** Summary statistics for Kruskal-Wallis tests comparing distribution of DuraClone immune cell subset counts between peak severity groups at baseline. Bonferroni adjustment is performed for 8 tests.

| Variable | p-value | Chi-squared | Bonferroni adj. p-value |
|---|---|---|---|
| NULL_IFNa | 0.998919157465403 | 0.0255967065004979 | 1 |
| NULL_IFNg | 0.194581278845731 | 4.7066560255387 | 1 |
| NULL_IL10 | 0.446761532133829 | 2.66177984288953 | 1 |
| NULL_IL12 | 0.327252363744088 | 3.45039936624089 | 1 |
| NULL_IL17A | 0.966894370783524 | 0.26264123827145 | 1 |
| NULL_IL1b | 0.974191041711318 | 0.220636570636572 | 1 |
| NULL_IL6 | 0.0791454888407777 | 6.78300094570539 | 0.712309399566999 |
| NULL_IL8 | 0.430403719183868 | 2.75834101382488 | 1 |
| NULL_TNFa | 0.44475378497575 | 2.67347495072796 | 1 |
| LPS_IFNa | 0.141164243674145 | 5.45813364055299 | 1 |
| LPS_IFNg | 0.293561805767592 | 3.71806217814228 | 1 |
| LPS_IL10 | 0.111068031010687 | 6.01117025081059 | 0.99961227909618 |
| LPS_IL12 | 0.316528963125271 | 3.53283087767817 | 1 |
| LPS_IL17A | 0.347769478527118 | 3.29908886044959 | 1 |
| LPS_IL1b | 0.134497885045118 | 5.57023041474653 | 1 |
| LPS_IL6 | 0.103357989225459 | 6.17592165898618 | 0.930221903029135 |
| LPS_IL8 | 0.466827419536634 | 2.54716589861751 | 1 |
| LPS_TNFa | 0.0490826006786499 | 7.85603686635945 | 0.441743406107849 |
| R848_IFNa | 0.0779728602868684 | 6.8167741935484 | 0.701755742581816 |
| R848_IFNg | 0.513899515894173 | 2.29281105990783 | 1 |
| R848_IL10 | 0.0803952128780934 | 6.74753456221197 | 0.723556915902841 |
| R848_IL12 | 0.319560884350235 | 3.50927725061988 | 1 |
| R848_IL17A | 0.810763098196414 | 0.960687843941844 | 1 |
| R848_IL1b | 0.313874401047154 | 3.55361751152074 | 1 |
| R848_IL6 | 0.104370950570321 | 6.15361751152074 | 0.939338555132889 |
| R848_IL8 | 0.884693033202135 | 0.650875576036867 | 1 |
| R848_TNFa | 0.243752680110454 | 4.16930875576035 | 1 |
| cd3_IFNa | 0.96393724058609 | 0.27895167895168 | 1 |
| cd3_IFNg | 0.880733465341969 | 0.667872046528527 | 1 |
| cd3_IL10 | 0.850406915124067 | 0.796069538926673 | 1 |
| cd3_IL12 | 0.757084071232035 | 1.18300151356385 | 1 |
| cd3_IL17A | 0.844569428042162 | 0.820451005226309 | 1 |
| cd3_IL1b | 0.867032746165558 | 0.726166484914575 | 1 |
| cd3_IL6 | 0.687636256714387 | 1.47679516250943 | 1 |
| cd3_IL8 | 0.620692152605392 | 1.77362055933484 | 1 |
| cd3_TNFa | 0.958306515926027 | 0.309107960741561 | 1 |
| poly_IFNa | 0.690289287474031 | 1.46535439499171 | 1 |
| poly_IFNg | 0.32511711833475 | 3.46662115550445 | 1 |
| poly_IL10 | 0.556289708914539 | 2.07842568902418 | 1 |
| poly_IL12 | 0.307559523050946 | 3.60370007103211 | 1 |
| poly_IL17A | 0.494144656911397 | 2.39726117954211 | 1 |
| poly_IL1b | 0.780885010796859 | 1.08421887582542 | 1 |
| poly_IL6 | 0.207523328922296 | 4.55400642554951 | 1 |
| poly_IL8 | 0.301900963470296 | 3.64936025812193 | 1 |
| poly_TNFa | 0.625597152635324 | 1.75126517896878 | 1 |

**Supplementary Table 5. Summary statistics from Kruskal-Wallis tests comparing cytokine concentrations at/near peak severity.** Summary statistics for Kruskal-Wallis tests comparing distribution of TruCulture cytokine concentrations between peak severity groups at/near peak severity. Bonferroni adjustment is performed within each stimulus (9 tests).

| Variable | p-value | Chi-squared | Bonferroni adj. p-value |
|---|---|---|---|
| Neutrophils | 0.228386046347443 | 4.32557787040545 | 1 |
| Eosinophils | 0.0788285850870587 | 6.79208033345964 | 0.630628680696469 |
| Monocytes | 0.124183240452981 | 5.75452823039031 | 0.993465923623845 |
| B-Cells | 0.998986084990091 | 0.0245235738802665 | 1 |
| T-Cells | 0.921149894840981 | 0.489693065555144 | 1 |
| CD4+ T-Cells | 0.963108654074823 | 0.283459643804463 | 1 |
| CD8+ T-Cells | 0.822299321595296 | 0.912959454338761 | 1 |
| NK-Cells | 0.676166351309935 | 1.52650625236831 | 1 |

**Supplementary Table 6. Summary statistics from Kruskal-Wallis tests comparing immune cell subset counts at/near peak severity.** Summary statistics for Kruskal-Wallis tests comparing distribution of DuraClone immune cell subset counts between peak severity groups at/near peak severity. Bonferroni adjustment is performed for 8 tests.

| Variable | p-value | Bonferroni adj. p-value |
|---|---|---|
| NULL_IFNa | 0.0832301704109573 | 0.749071533698616 |
| NULL_IFNg | 0.209286554781767 | 1 |
| NULL_IL10 | 0.55093480568048 | 1 |
| NULL_IL12 | 0.513655642510047 | 1 |
| NULL_IL17A | 0.451258974150342 | 1 |
| NULL_IL1b | 0.420965220516746 | 1 |
| NULL_IL6 | 0.932281494140625 | 1 |
| NULL_IL8 | 0.129737854003906 | 1 |
| NULL_TNFa | 0.420965220516746 | 1 |
| LPS_IFNa | 0.377822875976563 | 1 |
| LPS_IFNg | 0.010986328125 | 0.098876953125 |
| LPS_IL10 | 0.044769287109375 | 0.402923583984375 |
| LPS_IL12 | 0.007904052734375 | 0.071136474609375 |
| LPS_IL17A | 0.00975977204371061 | 0.0878379483933955 |
| LPS_IL1b | 0.0020904541015625 | 0.0188140869140625 |
| LPS_IL6 | 0.0201568603515625 | 0.181411743164063 |
| LPS_IL8 | 0.0395355224609375 | 0.355819702148438 |
| LPS_TNFa | 0.02667236328125 | 0.24005126953125 |
| R848_IFNa | 0.817581176757813 | 1 |
| R848_IFNg | 0.030517578125 | 0.274658203125 |
| R848_IL10 | 0.328948974609375 | 1 |
| R848_IL12 | 0.0031585693359375 | 0.0284271240234375 |
| R848_IL17A | 0.0714111328125 | 0.6427001953125 |
| R848_IL1b | 0.0149993896484375 | 0.134994506835938 |
| R848_IL6 | 0.10888671875 | 0.97998046875 |
| R848_IL8 | 0.0347900390625 | 0.3131103515625 |
| R848_TNFa | 0.088653564453125 | 0.797882080078125 |
| cd3_IFNa | 0.629260787503937 | 1 |
| cd3_IFNg | 0.29338289215179 | 1 |
| cd3_IL10 | 0.268066760405714 | 1 |
| cd3_IL12 | 0.0934924836898357 | 0.841432353208521 |
| cd3_IL17A | 0.576103543277185 | 1 |
| cd3_IL1b | 0.00317265532163019 | 0.0285538978946717 |
| cd3_IL6 | 0.49542236328125 | 1 |
| cd3_IL8 | 0.596588134765625 | 1 |
| cd3_TNFa | 0.07391357421875 | 0.66522216796875 |
| poly_IFNa | 0.0538558959960938 | 0.484703063964844 |
| poly_IFNg | 0.010479750627497 | 0.0943177556474733 |
| poly_IL10 | 0.58949792671724 | 1 |
| poly_IL12 | 0.414446222764492 | 1 |
| poly_IL17A | 0.0787915035643527 | 0.709123532079174 |
| poly_IL1b | 0.0582881833523563 | 0.524593650171207 |
| poly_IL6 | 0.129737854003906 | 1 |
| poly_IL8 | 0.0431671142578125 | 0.388504028320313 |
| poly_TNFa | 0.0665359497070313 | 0.598823547363281 |

**Supplementary Table 7. Summary statistics from Wilcoxon signed-rank tests comparing cytokine concentrations at discharge vs. baseline.** Summary statistics for Wilcoxon signed-rank tests comparing TruCulture cytokine concentrations at discharge vs. baseline. Bonferroni adjustment is performed within each stimulus (9 tests).

| Variable | p-value | Bonferroni adj. p-value |
|---|---|---|
| Neutrophils | 0.84375 | 1 |
| Eosinophils | 0.0078125 | 0.0625 |
| Monocytes | 0.109375 | 0.875 |
| B-Cells | 0.0759269629825577 | 0.607415703860461 |
| T-Cells | 0.015625 | 0.125 |
| CD4+ T-Cells | 0.015625 | 0.125 |
| CD8+ T-Cells | 0.0078125 | 0.0625 |
| NK-Cells | 0.109375 | 0.875 |

**Supplementary table 8. Summary statistics from Wilcoxon signed-rank tests comparing immune cell subset counts at discharge vs. baseline.** Summary statistics for Wilcoxon signed-rank tests comparing DuraClone immune cell subset counts at discharge vs. baseline. Bonferroni adjustment is performed for 8 tests.

| Variable | Estimate | Standard error | t-value | p-value | Multiple $r^2$ | Adjusted $r^2$ | F-statistic | numDF | denDF | Bonferroni adj. p-value |
|---|---|---|---|---|---|---|---|---|---|---|
| NULL_IFNa | -0.295683740704783 | 0.123355898832099 | -2.39699717244362 | 0.0264152932262636 | 0.331797100951949 | 0.264976811047144 | 4.96551423863382 | 2 | 20 | 1 |
| NULL_IFNg | 0.127701492053507 | 0.11378091063184 | 1.1223454913866 | 0.275009426840034 | 0.190801640395929 | 0.109881804435522 | 2.35790938193802 | 2 | 20 | 1 |
| NULL_IL10 | -0.11669255391038 | 0.152534478426062 | -0.765024112020319 | 0.453190561797869 | 0.164291345713833 | 0.0807204802852158 | 1.96589259751252 | 2 | 20 | 1 |
| NULL_IL12 | -0.0776146698570008 | 0.0766711290612142 | -1.01230633756591 | 0.323482595592759 | 0.181761021679033 | 0.0999371238469359 | 2.22136840818813 | 2 | 20 | 1 |
| NULL_IL17A | -0.145056294811518 | 0.136226376034539 | -1.06481798190638 | 0.299645571556467 | 0.185984010309814 | 0.104582411340795 | 2.28477097090684 | 2 | 20 | 1 |
| NULL_IL1b | -0.294340586076445 | 0.139740137804923 | -2.10634246323231 | 0.0480043373952788 | 0.29600573732275 | 0.225606311055025 | 4.20466121694028 | 2 | 20 | 1 |
| NULL_IL6 | 0.161528656344404 | 0.150662320263483 | 1.07212378026515 | 0.296431595884913 | 0.18658489928831 | 0.10524338921714 | 2.29384602185353 | 2 | 20 | 1 |
| NULL_IL8 | -0.195469946567918 | 0.153076456705842 | -1.27694323983172 | 0.216249278421297 | 0.20467790033576 | 0.125145690369336 | 2.57352210409052 | 2 | 20 | 1 |
| NULL_TNFa | -0.518853892434298 | 0.238971510793112 | -2.17119559863975 | 0.0421238317985008 | 0.303907961577308 | 0.234298757735039 | 4.36591635591678 | 2 | 20 | 1 |
| LPS_IFNa | -0.372312332176319 | 0.152653567221664 | -2.43893633769918 | 0.0241774606041096 | 0.337019832454896 | 0.270721815700385 | 5.08340745248564 | 2 | 20 | 1 |
| LPS_IFNg | -0.501510691134648 | 0.167846597582231 | -2.98791097560944 | 0.00727099696907206 | 0.405298940162827 | 0.345828834179109 | 6.81517097470444 | 2 | 20 | 0.327194863608243 |
| LPS_IL10 | -0.345548684626393 | 0.11672702478131 | -2.96031433400949 | 0.00773599394959747 | 0.401905023178544 | 0.342095525496399 | 6.71975252683858 | 2 | 20 | 0.348119727731886 |
| LPS_IL12 | -0.145773684290964 | 0.103079103717925 | -1.41419239237734 | 0.172684436202805 | 0.21803053017627 | 0.139833583193897 | 2.7882230520511 | 2 | 20 | 1 |
| LPS_IL17A | -0.610924788028871 | 0.168461699191816 | -3.62649071545485 | 0.00168157856254266 | 0.481069776890627 | 0.42917675457969 | 9.27041354438966 | 2 | 20 | 0.0756710353144199 |
| LPS_IL1b | -0.285703327638817 | 0.0625803900974216 | -4.565381059371 | 0.000187880085140405 | 0.578791810102595 | 0.536670991112855 | 13.7412287791359 | 2 | 20 | 0.00845460383131824 |
| LPS_IL6 | -0.422781727370785 | 0.122954526817099 | -3.4385210395685 | 0.00259965436149308 | 0.459414551847474 | 0.405356007032222 | 8.49846316465867 | 2 | 20 | 0.116984446267189 |
| LPS_IL8 | -0.470726970154452 | 0.188388302952899 | -2.49870593224753 | 0.0212922930149814 | 0.344475356852271 | 0.278922892537498 | 5.25495662829933 | 2 | 20 | 0.958153185674164 |
| LPS_TNFa | -0.252946104537422 | 0.0750619076292738 | -3.36983314874846 | 0.00304591396380602 | 0.45135206300098 | 0.396487269301078 | 8.2266246268923 | 2 | 20 | 0.137066128371271 |
| R848_IFNa | -0.257270616875665 | 0.0931702323824308 | -2.76129628849331 | 0.0120431002308961 | 0.377251304191618 | 0.314976434610779 | 6.05784173826189 | 2 | 20 | 0.541939510390323 |
| R848_IFNg | -0.45832562555849 | 0.161046162429588 | -2.84592702268754 | 0.00998696333393481 | 0.387768338171203 | 0.326545171988323 | 6.33368645151249 | 2 | 20 | 0.449413350027066 |
| R848_IL10 | -0.227626975821358 | 0.12049721848767 | -1.88906415167292 | 0.0734713809875036 | 0.270075089243783 | 0.197082598168162 | 3.70003935012959 | 2 | 20 | 1 |
| R848_IL12 | -0.653492474895523 | 0.150479555899346 | -4.3427326123466 | 0.000315725172402931 | 0.557293369582074 | 0.513022706540282 | 12.5883221820278 | 2 | 20 | 0.0142076327581319 |
| R848_IL17A | -0.605124861129442 | 0.156949698394175 | -3.85553376222289 | 0.000986046870776939 | 0.506576447937991 | 0.45723409273179 | 10.2665640061366 | 2 | 20 | 0.0443721091849623 |
| R848_IL1b | -0.270881655498033 | 0.0771812027794317 | -3.5096842980299 | 0.00220511397104436 | 0.467685397283704 | 0.414453937012074 | 8.78588328964108 | 2 | 20 | 0.0992301286969961 |
| R848_IL6 | -0.468770098107008 | 0.13323372461837 | -3.51840421372093 | 0.00216102009387362 | 0.468692913860721 | 0.415562205246793 | 8.82150692298231 | 2 | 20 | 0.0972459042243131 |
| R848_IL8 | -0.508002075411165 | 0.192335354989087 | -2.6412308617934 | 0.0156622073522764 | 0.362276933391315 | 0.298504626730447 | 5.68078767038881 | 2 | 20 | 0.70479933085244 |
| R848_TNFa | -0.228841165581337 | 0.0889565326379457 | -2.57250545626282 | 0.0181745301391156 | 0.353692204225825 | 0.289061424648407 | 5.47250406908921 | 2 | 20 | 0.817853856260201 |
| cd3_IFNa | -0.155102674905774 | 0.142907336558934 | -1.08533738463323 | 0.292094855114466 | 0.258392003057555 | 0.175991114508394 | 3.13579146544515 | 2 | 18 | 1 |
| cd3_IFNg | 0.0192828801654031 | 0.0946826666019468 | 0.203657975186418 | 0.840905505366104 | 0.211676139853599 | 0.124084599837333 | 2.41662767676295 | 2 | 18 | 1 |
| cd3_IL10 | -0.164961774038078 | 0.135655699288823 | -1.21603275721471 | 0.239679881532924 | 0.269843448953822 | 0.188714943282025 | 3.3261237430585 | 2 | 18 | 1 |
| cd3_IL12 | -0.0770471622352413 | 0.0796585260817262 | -0.967218024548864 | 0.346252065200855 | 0.248896601040078 | 0.165440667822309 | 2.9823715515901 | 2 | 18 | 1 |
| cd3_IL17A | -0.00743182651274496 | 0.121576847783472 | -0.0611286330270795 | 0.951930393859608 | 0.210023636628931 | 0.122248485143257 | 2.3927459317824 | 2 | 18 | 1 |
| cd3_IL1b | -0.0383503480922871 | 0.104515413360152 | -0.366934855437397 | 0.717945790784779 | 0.21572606231744 | 0.128584513686045 | 2.47558215002525 | 2 | 18 | 1 |
| cd3_IL6 | -0.0631605771194358 | 0.162902397955604 | -0.387720364537844 | 0.702769720457326 | 0.216403848286207 | 0.129337609206896 | 2.4855081668232 | 2 | 18 | 1 |
| cd3_IL8 | -0.12084541632915 | 0.124394164986245 | -0.971471743409453 | 0.344187921974833 | 0.249223573147279 | 0.165803970163643 | 2.98759001761455 | 2 | 18 | 1 |
| cd3_TNFa | -0.0231338809458893 | 0.102670046471575 | -0.225322591553459 | 0.824266698568166 | 0.212082019762442 | 0.124535577513824 | 2.42250872011639 | 2 | 18 | 1 |
| poly_IFNa | -0.139107973756631 | 0.1318948867193 | -1.05468814763594 | 0.304143173585663 | 0.185156195819253 | 0.103671815401178 | 2.27229065091083 | 2 | 20 | 1 |
| poly_IFNg | 0.15724762533771 | 0.128832391926487 | 1.22055969765303 | 0.236448622272913 | 0.199466316503688 | 0.119412948154056 | 2.4916667545146 | 2 | 20 | 1 |
| poly_IL10 | -0.155248478134798 | 0.156545436589049 | -0.991715130875036 | 0.333183997820598 | 0.180151909117147 | 0.0981671000288618 | 2.19738157739869 | 2 | 20 | 1 |
| poly_IL12 | -0.0713324426147451 | 0.0805645713798779 | -0.885407088910068 | 0.386458874660382 | 0.172280290213038 | 0.0895083192343421 | 2.08138441281506 | 2 | 20 | 1 |
| poly_IL17A | -0.115327006573926 | 0.141572085152071 | -0.814616853668901 | 0.424885893970828 | 0.167459639214638 | 0.0842056031361016 | 2.01142968079851 | 2 | 20 | 1 |
| poly_IL1b | -0.246163980678001 | 0.147364831438895 | -1.67043912902702 | 0.110409083171143 | 0.245151181567469 | 0.169666299724216 | 3.24768583564234 | 2 | 20 | 1 |
| poly_IL6 | 0.114925151323175 | 0.15100959334271 | 0.76104536658381 | 0.455510123489135 | 0.164044771826686 | 0.0804492490093546 | 1.96236313020194 | 2 | 20 | 1 |
| poly_IL8 | -0.226386150595032 | 0.179351092418611 | -1.26225130576088 | 0.221379535299336 | 0.203303860213427 | 0.123634246234769 | 2.55183689314586 | 2 | 20 | 1 |
| poly_TNFa | -0.53241388475189 | 0.207312657454275 | -2.56816873262705 | 0.0183451766798455 | 0.3531504363945 | 0.28846548003395 | 5.45954509772041 | 2 | 20 | 0.825532950593047 |

**Supplementary Table 9. Summary statistics from all ordinary least squares analyses at baseline.** Summary statistics for associations between each stimulus-response variable at baseline and future peak severity after adjusting for age. Bonferroni adjustment is performed for 45 tests. numDF, degrees of freedom in the numerator; denDF, degrees of freedom in the denominator.

| Variable | Estimate | Standard error | t-value | p-value | Multiple $r^2$ | Adjusted $r^2$ | F-statistic | numDF | denDF | Bonferroni adj. p-value |
|---|---|---|---|---|---|---|---|---|---|---|
| NULL_IFNa | -0.121342936769864 | 0.110794692962403 | -1.09520531647703 | 0.283100503425482 | 0.192980588691204 | 0.133201373038701 | 3.22822216024045 | 2 | 27 | 1 |
| NULL_IFNg | 0.148347558717363 | 0.0794486922275139 | 1.86721209069806 | 0.0727650587852438 | 0.253520815802481 | 0.19822606141748 | 4.58489812681487 | 2 | 27 | 1 |
| NULL_IL10 | -0.158339340657195 | 0.109052986433793 | -1.45194868875346 | 0.158038024651502 | 0.218173619599525 | 0.160260554384675 | 3.76726078632048 | 2 | 27 | 1 |
| NULL_IL12 | 0.0258264204468998 | 0.0513933531816741 | 0.502524525994717 | 0.619372875085219 | 0.164939082397464 | 0.103082718130609 | 2.66648524129062 | 2 | 27 | 1 |
| NULL_IL17A | -0.00296815595575835 | 0.0944674141094774 | -0.0314198920732453 | 0.975165791342303 | 0.157159577795253 | 0.0947269539282352 | 2.51726690407893 | 2 | 27 | 1 |
| NULL_IL1b | -0.00530470174983513 | 0.0853856455203617 | -0.0621263880773745 | 0.950919813047794 | 0.157249233163073 | 0.0948232504344112 | 2.51897088823682 | 2 | 27 | 1 |
| NULL_IL6 | 0.236750796755912 | 0.101183403308118 | 2.33981847828315 | 0.0269364769400111 | 0.299224070966463 | 0.247314742889904 | 5.76436031930818 | 2 | 27 | 1 |
| NULL_IL8 | 0.128604780206232 | 0.103290573392693 | 1.24507760952491 | 0.223801539906883 | 0.202894939105486 | 0.143850119779966 | 3.43628689905726 | 2 | 27 | 1 |
| NULL_TNFa | 0.0914264861539444 | 0.161215942620501 | 0.56710573822814 | 0.575327407841354 | 0.167050391222375 | 0.10535042020181 | 2.70746304186588 | 2 | 27 | 1 |
| LPS_IFNa | -0.318445305638024 | 0.116895816489846 | -2.72418051561055 | 0.0111659407774574 | 0.338850759172038 | 0.28987674133293 | 6.9189903978319 | 2 | 27 | 0.502467334985583 |
| LPS_IFNg | -0.248974586424172 | 0.128523920307725 | -1.93718481219723 | 0.0632502380297901 | 0.259982552345926 | 0.20516644511229 | 4.74281311582083 | 2 | 27 | 1 |
| LPS_IL10 | -0.228177261440332 | 0.0773547349154288 | -2.94975170802144 | 0.00649652830371202 | 0.362552839963609 | 0.315334531812765 | 7.67822597127783 | 2 | 27 | 0.292343773667041 |
| LPS_IL12 | -0.35692672132189 | 0.147155119334442 | -2.42551345095033 | 0.0222479265985108 | 0.307926785125135 | 0.256662102541812 | 6.00660668530709 | 2 | 27 | 1 |
| LPS_IL17A | -0.304091460549907 | 0.104624270442242 | -2.90650973492602 | 0.00721636613008468 | 0.35799896735703 | 0.310443335309403 | 7.52800356009339 | 2 | 27 | 0.324736475853811 |
| LPS_IL1b | -0.240605490728191 | 0.0746420442675019 | -3.22345794638086 | 0.00329958145205506 | 0.39135841416458 | 0.346273852250845 | 8.68054157681311 | 2 | 27 | 0.148481165342478 |
| LPS_IL6 | -0.266624025189833 | 0.101831313751743 | -2.61829112643918 | 0.0143126278355805 | 0.327803218687251 | 0.278010864515937 | 6.58340470425273 | 2 | 27 | 0.644068252601123 |
| LPS_IL8 | -0.331519679385443 | 0.223962052626971 | -1.48024933463893 | 0.150382035379019 | 0.220396156277607 | 0.162647723409281 | 3.81648722451293 | 2 | 27 | 1 |
| LPS_TNFa | -0.252076723966594 | 0.0775226600038183 | -3.25165214859988 | 0.00307375669575708 | 0.394315676988063 | 0.349450171579771 | 8.78883840490937 | 2 | 27 | 0.138319051309068 |
| R848_IFNa | -0.365535895058894 | 0.105348389184675 | -3.46978153048084 | 0.00176531394947774 | 0.41706243274973 | 0.373881872212673 | 9.65856921639108 | 2 | 27 | 0.0794391277264982 |
| R848_IFNg | -0.1894110364423 | 0.127399319738143 | -1.48675076783468 | 0.148666096907452 | 0.220910984941877 | 0.163200687530164 | 3.82793010692474 | 2 | 27 | 1 |
| R848_IL10 | -0.179889008884164 | 0.116367310657043 | -1.54587235769615 | 0.133777302230961 | 0.225663857388943 | 0.168305624602939 | 3.93428887934649 | 2 | 27 | 1 |
| R848_IL12 | -0.303895128836472 | 0.136800881450856 | -2.22144130661646 | 0.0348963293224011 | 0.287375452299323 | 0.23458844876594 | 5.4440569280956 | 2 | 27 | 1 |
| R848_IL17A | -0.0705184010841038 | 0.130368918989449 | -0.540914211997195 | 0.593001828207664 | 0.166164699893754 | 0.104399122108106 | 2.69024764036717 | 2 | 27 | 1 |
| R848_IL1b | -0.202484655436996 | 0.0887625915049529 | -2.28119359748186 | 0.0306453384238026 | 0.293328979679078 | 0.240982978173825 | 5.60365589050081 | 2 | 27 | 1 |
| R848_IL6 | -0.284170547549882 | 0.121597413223348 | -2.33697855914025 | 0.0271062705100021 | 0.298937346002118 | 0.247006779039311 | 5.75648146141412 | 2 | 27 | 1 |
| R848_IL8 | 0.000308289158217061 | 0.189682235023834 | 0.00162529273328271 | 0.99871515384421 | 0.157128843215159 | 0.0946939427125786 | 2.51668284805971 | 2 | 27 | 1 |
| R848_TNFa | -0.25809369685082 | 0.104018403507492 | -2.48123109130618 | 0.0196145278960591 | 0.313633279895691 | 0.262791300628705 | 6.16878580294273 | 2 | 27 | 0.882653755322661 |
| cd3_IFNa | -0.0380862658745402 | 0.102782575044448 | -0.370551777459069 | 0.714221840699237 | 0.207996944640394 | 0.141996690027093 | 3.15145670056972 | 2 | 24 | 1 |
| cd3_IFNg | 0.010591795197176 | 0.0672529463375294 | 0.157491913350797 | 0.876174707283397 | 0.204288101100399 | 0.137978776192099 | 3.08083518242586 | 2 | 24 | 1 |
| cd3_IL10 | -0.0771146341942363 | 0.106161029146351 | -0.726393054158581 | 0.474623315577906 | 0.220601051661333 | 0.155651139299777 | 3.39647958927668 | 2 | 24 | 1 |
| cd3_IL12 | -0.0203337613893853 | 0.0631174082387085 | -0.322157736776573 | 0.750122646215754 | 0.206895444291506 | 0.140803397982465 | 3.13041365558694 | 2 | 24 | 1 |
| cd3_IL17A | -0.0062455173005642 | 0.075840959182301 | -0.0823501887093974 | 0.935051214909252 | 0.203690751617189 | 0.137331647585288 | 3.06952232988662 | 2 | 24 | 1 |
| cd3_IL1b | -0.0171556635084759 | 0.0813106662016466 | -0.210989090483291 | 0.834677023884578 | 0.204940458774984 | 0.138685497006233 | 3.09320922243205 | 2 | 24 | 1 |
| cd3_IL6 | 0.0529800647984658 | 0.117992511386293 | 0.449012095564399 | 0.657451273254772 | 0.210101282341972 | 0.144276389203803 | 3.19182109268239 | 2 | 24 | 1 |
| cd3_IL8 | 0.028609230293433 | 0.109792627494471 | 0.260575149227335 | 0.796642906847233 | 0.205712893732883 | 0.139522301543957 | 3.10788719257445 | 2 | 24 | 1 |
| cd3_TNFa | 0.00232211877731146 | 0.0766124065172257 | 0.0303099573929889 | 0.976070505191226 | 0.203496231992753 | 0.137120917992149 | 3.06584209893005 | 2 | 24 | 1 |
| poly_IFNa | -0.22122915573443 | 0.0921115985386394 | -2.4017513455879 | 0.023466786127382 | 0.305504179517187 | 0.254060044666609 | 5.93856190612467 | 2 | 27 | 1 |
| poly_IFNg | 0.10621555942671 | 0.0799657764645344 | 1.32826271591094 | 0.195210396617638 | 0.208826989516874 | 0.150221581332939 | 3.56327164997235 | 2 | 27 | 1 |
| poly_IL10 | -0.163014915913594 | 0.109246275970687 | -1.49217824099865 | 0.1472457350594 | 0.221341974436594 | 0.163663602172638 | 3.83752116692296 | 2 | 27 | 1 |
| poly_IL12 | 0.0486673122142006 | 0.050561845826214 | 0.962530370854635 | 0.344325079282928 | 0.185091142323348 | 0.124727523236188 | 3.06626980161832 | 2 | 27 | 1 |
| poly_IL17A | -0.0863929682240232 | 0.0933432770001292 | -0.925540338849509 | 0.362884085225318 | 0.183048071113962 | 0.1225331134187 | 3.02484010706485 | 2 | 27 | 1 |
| poly_IL1b | 0.0222115314206228 | 0.0923563595021481 | 0.240498126391677 | 0.811760870458535 | 0.158930498243558 | 0.0966290536690065 | 2.55099218531567 | 2 | 27 | 1 |
| poly_IL6 | 0.172684901323523 | 0.106119838732169 | 1.62726313370448 | 0.115296828422891 | 0.232409108930145 | 0.175550524406452 | 4.08749375097968 | 2 | 27 | 1 |
| poly_IL8 | 0.135507603999747 | 0.119057673278044 | 1.13816774903106 | 0.265049480350093 | 0.195717230039175 | 0.136140728560596 | 3.28514137590883 | 2 | 27 | 1 |
| poly_TNFa | -0.0129385420742466 | 0.173707780877067 | -0.0744845280327613 | 0.941173969611373 | 0.157301917883012 | 0.0948798377261977 | 2.51997237977083 | 2 | 27 | 1 |

**Supplementary Table 10. Summary statistics from all ordinary least squares analyses at/near peak severity.** Summary statistics for associations between each stimulus-response variable at/near peak severity and current severity after adjusting for age. Bonferroni adjustment is performed for 45 tests. numDF, degrees of freedom in the numerator; denDF, degrees of freedom in the denominator.

| Variable | Estimate | Standard error | t-value | p-value | Multiple $r^2$ | Adjusted $r^2$ | F-statistic | numDF | denDF | Bonferroni adj. p-value |
|---|---|---|---|---|---|---|---|---|---|---|
| NULL_IFNa | 0.0583472221381673 | 0.123430149758539 | 0.472714504943156 | 0.64107291044527 | 0.0595061949749823 | -0.0259932418454738 | 0.695983472966516 | 2 | 22 | 1 |
| NULL_IFNg | 0.103761600004828 | 0.0864733243147798 | 1.19992611394371 | 0.242930917404724 | 0.108311278416161 | 0.0272486673630844 | 1.33614346995612 | 2 | 22 | 1 |
| NULL_IL10 | 0.107981973959869 | 0.124247792120771 | 0.869085656306146 | 0.39418319394005 | 0.081487985407238 | -0.00201310682846745 | 0.97589125154453 | 2 | 22 | 1 |
| NULL_IL12 | 0.0916186942995399 | 0.0493132448120427 | 1.85789222852287 | 0.0766191995766349 | 0.178798534263388 | 0.104143855560059 | 2.39500775261412 | 2 | 22 | 1 |
| NULL_IL17A | 0.192383023275375 | 0.0857543826295573 | 2.24341913936263 | 0.035266052948652 | 0.226830907501645 | 0.156542808183612 | 3.22715950071866 | 2 | 22 | 1 |
| NULL_IL1b | 0.136692223156853 | 0.0651454599594219 | 2.0982616937849 | 0.0475857116090602 | 0.208375513560188 | 0.136409651156569 | 2.89547719711212 | 2 | 22 | 1 |
| NULL_IL6 | 0.0912078920819798 | 0.0804978103464916 | 1.13304811260565 | 0.269391622279202 | 0.102336075246405 | 0.0207302639051694 | 1.25402926047124 | 2 | 22 | 1 |
| NULL_IL8 | 0.149911098992227 | 0.0834510877530998 | 1.79639478679723 | 0.0861778316541191 | 0.171483047585536 | 0.0961633246387671 | 2.27673497560165 | 2 | 22 | 1 |
| NULL_TNFa | 0.130499609189783 | 0.107151156648793 | 1.21790201124491 | 0.236162681558103 | 0.109961607002393 | 0.0290490258207925 | 1.35901741603812 | 2 | 22 | 1 |
| LPS_IFNa | -0.0459908889718324 | 0.284230840450057 | -0.161808229180934 | 0.873003586682615 | 0.0493814176059756 | -0.0411536854791696 | 0.545439458543876 | 2 | 21 | 1 |
| LPS_IFNg | 0.0877469990986169 | 0.247038631091895 | 0.355195455507427 | 0.725985815019896 | 0.0538803252661282 | -0.036226310422812 | 0.597961791095276 | 2 | 21 | 1 |
| LPS_IL10 | -0.0569321805541318 | 0.182481177254696 | -0.311989331780063 | 0.758125197891279 | 0.0525875891958078 | -0.0376421642141154 | 0.582818717866789 | 2 | 21 | 1 |
| LPS_IL12 | -0.0433993401752628 | 0.299357564051735 | -0.144974924260683 | 0.886113096495116 | 0.0491478818096276 | -0.0414094627799317 | 0.542726622919264 | 2 | 21 | 1 |
| LPS_IL17A | -0.00465683427399524 | 0.279911320216566 | -0.0166368200842762 | 0.986883403160056 | 0.048208771740877 | -0.0424380119028491 | 0.531831023705757 | 2 | 21 | 1 |
| LPS_IL1b | -0.0873070425819999 | 0.160828822578615 | -0.542856940579312 | 0.592943871935681 | 0.0613680781282459 | -0.0280254382404928 | 0.686493614090638 | 2 | 21 | 1 |
| LPS_IL6 | -0.20477648247948 | 0.19082643293247 | -1.07310333968223 | 0.295401832908633 | 0.0976758496747576 | 0.0117402163104487 | 1.13661639358237 | 2 | 21 | 1 |
| LPS_IL8 | -0.108750422295639 | 0.41661785361646 | -0.261031593705427 | 0.796610940967028 | 0.0512745020511615 | -0.0390803072772994 | 0.567479500341445 | 2 | 21 | 1 |
| LPS_TNFa | -0.161571270208186 | 0.140877378926783 | -1.14689293227238 | 0.264325706558798 | 0.104299630830883 | 0.018994833767157 | 1.22267017120933 | 2 | 21 | 1 |
| R848_IFNa | -0.186434649769571 | 0.159396871404104 | -1.16962552732243 | 0.25525611418886 | 0.106408355683825 | 0.0213043895584754 | 1.2503336862949 | 2 | 21 | 1 |
| R848_IFNg | 0.0361963180585161 | 0.172965898305367 | 0.209268522946716 | 0.836255344250479 | 0.0501769840203181 | -0.0402823508348897 | 0.554691056491108 | 2 | 21 | 1 |
| R848_IL10 | -0.0687948675248487 | 0.19669591620189 | -0.349752393711303 | 0.730007752583351 | 0.0537084534871185 | -0.0364145509426799 | 0.59594610529163 | 2 | 21 | 1 |
| R848_IL12 | -0.0231936404690635 | 0.258173294277551 | -0.0898374889392279 | 0.929267687478415 | 0.0485618861225118 | -0.0420512675801064 | 0.535925350108511 | 2 | 21 | 1 |
| R848_IL17A | 0.117936224161378 | 0.212237132733196 | 0.555681386393567 | 0.584300871943463 | 0.0619886477587837 | -0.0273457667403798 | 0.693894375491364 | 2 | 21 | 1 |
| R848_IL1b | 0.0720350871701272 | 0.175361748582335 | 0.410779932068854 | 0.685395250722721 | 0.0557832402996397 | -0.0341421653861091 | 0.62032792484227 | 2 | 21 | 1 |
| R848_IL6 | -0.161608271762704 | 0.195912350752824 | -0.824900886246832 | 0.418699888275412 | 0.0780694746967741 | -0.00973343247496183 | 0.889144530762247 | 2 | 21 | 1 |
| R848_IL8 | 0.266648332111075 | 0.294602815855559 | 0.905111281223496 | 0.37567399570702 | 0.0839327280157287 | -0.00331177407801153 | 0.962040312013552 | 2 | 21 | 1 |
| R848_TNFa | -0.100783223444001 | 0.185225584243924 | -0.544110706171558 | 0.592096132730467 | 0.06142814418128 | -0.027959651610979 | 0.687209519340219 | 2 | 21 | 1 |
| cd3_IFNa | 0.179059946206097 | 0.129940463426672 | 1.37801529626793 | 0.182706034009221 | 0.151125252252749 | 0.0702800381815822 | 1.86931600081751 | 2 | 21 | 1 |
| cd3_IFNg | 0.0537920958928889 | 0.0684153888831328 | 0.786257255436735 | 0.440499431902989 | 0.10083541919158 | 0.0152006972098256 | 1.17750623646638 | 2 | 21 | 1 |
| cd3_IL10 | 0.0737469468592045 | 0.0888486327293967 | 0.830029057214792 | 0.415858966751162 | 0.103768428342278 | 0.0184130405653524 | 1.21572206564716 | 2 | 21 | 1 |
| cd3_IL12 | 0.0547265613714078 | 0.0725504303012021 | 0.754324421567228 | 0.459030281086998 | 0.0987845816294005 | 0.0129545417845814 | 1.15093249179429 | 2 | 21 | 1 |
| cd3_IL17A | 0.0758918433119079 | 0.0662606735544514 | 1.14535272946693 | 0.264948718620176 | 0.128788734032584 | 0.0458162325118782 | 1.55218574433898 | 2 | 21 | 1 |
| cd3_IL1b | 0.126027009925793 | 0.0720842801743891 | 1.7483286178471 | 0.0950128134573598 | 0.191977362746209 | 0.115022825864896 | 2.49468544060365 | 2 | 21 | 1 |
| cd3_IL6 | 0.137437211199782 | 0.0810888753258084 | 1.69489600944114 | 0.104874281067081 | 0.185750142595809 | 0.108202537128743 | 2.39530468383962 | 2 | 21 | 1 |
| cd3_IL8 | 0.139112925612377 | 0.096508554961205 | 1.44145693268641 | 0.16420252330136 | 0.157704730312418 | 0.0774861331993145 | 1.96593727624112 | 2 | 21 | 1 |
| cd3_TNFa | 0.0760064607404361 | 0.0733781254427086 | 1.03581905754433 | 0.31206781323195 | 0.119358962081166 | 0.0354883870412772 | 1.42313274976831 | 2 | 21 | 1 |
| poly_IFNa | 0.0671749084523464 | 0.166504991169162 | 0.403440809675786 | 0.690518314777513 | 0.0569305769113455 | -0.0288030070058047 | 0.664040558089359 | 2 | 22 | 1 |
| poly_IFNg | 0.235002023870212 | 0.0956124719675303 | 2.45785951387198 | 0.0223239771116143 | 0.254628193861265 | 0.186867120575925 | 3.75773554809314 | 2 | 22 | 1 |
| poly_IL10 | 0.112537810763753 | 0.134555273994743 | 0.836368634410796 | 0.411940268650269 | 0.0792302026084802 | -0.00447614260893059 | 0.94652564752045 | 2 | 22 | 1 |
| poly_IL12 | 0.0811481416525758 | 0.0477945669333474 | 1.69785284937809 | 0.103637769239668 | 0.160017927782755 | 0.0836559212175512 | 2.09551758761235 | 2 | 22 | 1 |
| poly_IL17A | 0.129705695410906 | 0.119283667169472 | 1.08737179606179 | 0.288642613364376 | 0.09840888458223 | 0.0164460559078873 | 1.20065261501932 | 2 | 22 | 1 |
| poly_IL1b | 0.159941782269611 | 0.0751091794033937 | 2.12945719205107 | 0.0446504969276294 | 0.212310152632792 | 0.140701984690318 | 2.9648873687615 | 2 | 22 | 1 |
| poly_IL6 | 0.0974960465830694 | 0.135056955733248 | 0.721888377046159 | 0.477966394499793 | 0.0719367911848805 | -0.0124325914346757 | 0.852640957552841 | 2 | 22 | 1 |
| poly_IL8 | 0.172306042929773 | 0.0873918185618281 | 1.97164958648697 | 0.0613541169655031 | 0.19261788245387 | 0.119219508131495 | 2.62427995486476 | 2 | 22 | 1 |
| poly_TNFa | 0.200898602583441 | 0.158511619194819 | 1.26740615990129 | 0.218261828489363 | 0.114600371500361 | 0.0341094961822122 | 1.42376848366216 | 2 | 22 | 1 |

**Supplementary Table 11. Summary statistics from all ordinary least squares analyses at discharge.** Summary statistics for associations between each stimulus-response variable and previous pear severity at discharge after adjusting for age. Bonferroni adjustment is performed for 45 tests. numDF, degrees of freedom in the numerator; denDF, degrees of freedom in the denominator.

| | Grade 1 | Grade 2 | Grade 3 | Grade 4 | All |
|---|---|---|---|---|---|
| Patients, n | 15 | 5 | 6 | 4 | 30 |
| Median age, years | 68 | 74 | 77 | 73 | 70 |
| Sex - Male, n (%) | 10 (66,7) | 4 (80) | 2 (33,3) | 4 (100) | 20 (66,7) |
| Smoking, n (%) | | | | | |
| *Yes* | 2 (13,3) | 1 (20) | 0 | 1 (25) | 5 (16,7) |
| *Previous* | 8 (53,3) | 3 (60) | 2 (33,3) | 2 (50) | 15 (50) |
| Median BMI, $kg/m^2$ | 24 | 26,3 | 26,2 | 23 | 24,5 |
| Outcome, n (%) | | | | | |
| *ICU* | 0 | 0 | 3 (50) | 2 (50) | 5 (16,7) |
| *Death* | 0 | 0 | 0 | 2 (50) | 2 (6,7) |
| Days hospitalized, median | 6 | 16 | 10 | 28 | 8 |
| Days with symptoms prior to hospitalization, median | 5 | 5 | 6,5 | 8 | 6 |
| Immune suppressive precondition present, n (%) | 9 (60) | 2 (40) | 3 (50) | 3 (75) | 17 (56,7) |

**Supplementary Table 12. Baseline patient characteristics.** BMI, body mass index; ICU, intensive care unit.

| Subject | Peak Severity Grade | Age (years) | Sex | Smoking | BMI (kg/m$^2$) | Days hospitalized | Days to peak severity sample | Immunosuppressive precondition | Dexamethasone prior to baseline sample | Dexamethasone during hospitalization | At least 1 comorbidity | At least 2 comorbidities |
|---|---|---|---|---|---|---|---|---|---|---|---|---|
| **1** | 1 | 86 | M | Never | 22.4 | 3 | 1 | No | No | No | Yes | Yes |
| **2** | 3 | 57 | M | Previous | 26.3 | 51 | 23 | Yes | No | No | Yes | No |
| **3** | 2 | 85 | M | Yes | 20.8 | 38 | 18 | Yes | No | No | Yes | No |
| **4** | 1 | 64 | M | Yes | 22.8 | 24 | 17 | Yes | Yes | Yes | Yes | No |
| **5** | 2 | 65 | M | Previous | 32.3 | 16 | 9 | No | No | No | Yes | No |
| **6** | 1 | 28 | F | Previous | 23.4 | 36 | 29 | Yes | No | No | No | No |
| **7** | 1 | 65 | M | Previous | 35.3 | 8 | 6 | Yes | No | No | Yes | No |
| **8** | 4 | 77 | M | Yes | 21.1 | 10 | 7 | Yes | No | No | Yes | No |
| **9** | 1 | 26 | F | NA | NA | 4 | 2 | No | No | No | Yes | No |
| **10** | 4 | 69 | M | NA | 26.1 | 50 | 17 | Yes | No | No | No | No |
| **11** | 2 | 67 | F | Yes | 24.1 | 26 | 14 | Yes | No | No | Yes | No |
| **12** | 4 | 74 | M | Previous | 23.9 | 8 | 3 | Yes | No | No | Yes | No |
| **13** | 1 | 77 | M | Previous | 22.6 | 5 | 5 | Yes | No | No | Yes | Yes |
| **14** | 1 | 53 | F | Never | 23.6 | 2 | 2 | No | No | No | No | No |
| **15** | 1 | 74 | M | Previous | 26.7 | 6 | 6 | Yes | No | No | Yes | Yes |
| **16** | 1 | 36 | M | Yes | 15 | 29 | 11 | Yes | No | No | No | No |
| **17** | 1 | 69 | M | Never | 24.5 | 3 | 2 | Yes | No | No | Yes | Yes |
| **18** | 3 | 29 | F | Never | 43.1 | 6 | 5 | No | Yes | Yes | Yes | No |
| **19** | 4 | 72 | M | Previous | 22 | 47 | 18 | No | Yes | Yes | Yes | No |
| **20** | 2 | 74 | M | Previous | 30.6 | 7 | 3 | No | Yes | Yes | Yes | Yes |
| **21** | 1 | 50 | M | Previous | 26.6 | 9 | 7 | Yes | No | No | Yes | No |
| **22** | 1 | 68 | F | Previous | NA | 7 | 5 | No | Yes | Yes | Yes | Yes |
| **23** | 3 | 89 | F | Previous | 26 | 6 | 6 | Yes | No | No | Yes | Yes |
| **24** | 1 | 70 | M | Previous | 28.6 | 4 | 1 | No | No | Yes | Yes | Yes |
| **25** | 3 | 86 | M | Never | 23.4 | 19 | 5 | No | No | Yes | Yes | Yes |
| **26** | 3 | 81 | F | Never | 21.3 | 13 | 6 | No | No | Yes | Yes | No |
| **27** | 1 | 86 | M | Never | NA | 13 | 1 | Yes | No | No | Yes | No |
| **28** | 1 | 57 | F | Previous | 25.4 | 4 | 2 | No | No | No | Yes | No |
| **29** | 3 | 85 | F | Never | 27.7 | 7 | 1 | Yes | No | No | Yes | Yes |
| **30** | 2 | 64 | M | Previous | 26.3 | 11 | 2 | No | Yes | Yes | Yes | Yes |

**Supplementary Table 13. Detailed overview of clinical patient characteristics.** Days hospitalized indicate total number of days in hospital due to COVID-19. Days to peak severity sample indicate number of days from hospitalization to collection of peak severity sample. BMI, body mass index; F, female; M, male; NA, data not available.

| Covariate | p-value (Baseline) | p-value (At/near peak severity) | p-value (Discharge) |
|---|---|---|---|
| Age | 0.0787835405518851 | 0.160284869086185 | 0.282840876474055 |
| Sex | 0.694069392458504 | 0.82367807353201 | 0.445162998222237 |
| Days in hospital | 0.57709608150512 | 0.300927379241451 | 0.110246511862609 |
| Immune suppression | 0.729632149958679 | 0.857040205726494 | 0.325993590745308 |
| Admission hospital (1) | 0.400692966174122 | 0.404446269254582 | 0.455564995672469 |
| Admission hospital (2) | 0.445840745895935 | 0.424969618219765 | 0.349416275864357 |
| Admission hospital (3) | NA | NA | NA |

**Supplementary Table 14. P-values for associations between peak severity and covariates.** Associations between peak severity and covariates at baseline (n=23), at/near peak severity (n=30), and discharge (n=25), assessed by univariate linear regression analyses for "Age", "Sex", "Days in hospital" (days from hospitalization to sample taken), and "Immune suppression" (presence of an immunosuppressive precondition), and by multivariate linear regression analyses for "Admission hospital (1-3)". Due to only one observation from Admission hospital 3, p-values for this variable were not available (NA).

| | **Original cohort** | **Validation cohort** |
|---|---|---|
| **Patients, n** | 23 (Baseline samples) | 20 |
| **Grade 1** | 11 | 5 |
| **Grade 2** | 3 | 5 |
| **Grade 3** | 5 | 5 |
| **Grade 4** | 4 | 5 |
| **Age - median** | 70,5 | 69 |
| **Sex – male (%)** | 66,7 | 70 |
| **Admission hospital (1) (%)** | 73 | 75 |
| **Immune suppressive precondition present (%)** | 56,7 | 30 |
| **Even distribution of immune suppressive precondition across severity groups** | Yes | Yes |
| **Inclusion in COVIMUN study** | April 2020 - October 2020 | October 2020 – January 2021 |

**Supplementary Table 15. Baseline patient characteristics of the validation vs the original cohort.**

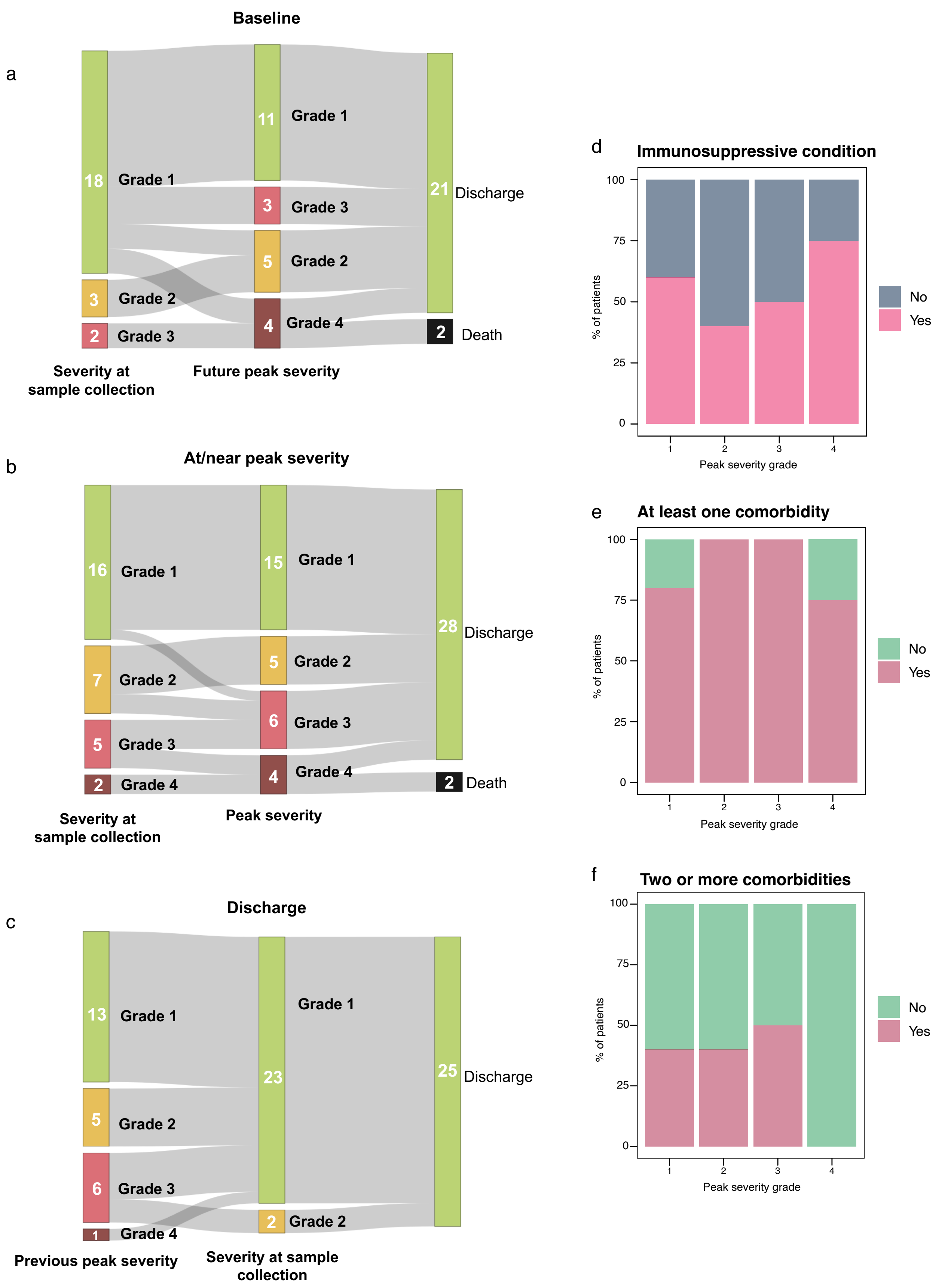


**Supplementary Figure 1. Severity at time of sample collection with corresponding peak severity and outcome, and distribution of immunosuppressive conditions and comorbidities across severity groups. a-c**, Sankey plots illustrating severity grade at time of sample collection with corresponding peak severity for (**a**) baseline samples (n=23), (**b**) samples collected at/near peak severity (n=30), and (**c**) samples collected at discharge (n=25). **d-f**, Percentage of patients (n=30) across peak severity groups with (**d**) a pre-existing immunosuppressive condition, (**e**) at least one comorbidity, (**f**) two or more comorbidities. The percentage of patients is presented on the y-axis. The x-axis shows the groups of patients with peak severity grade 1 (1; n=15), grade 2 (2; n=5), grade 3 (3; n=6), and grade 4 (4; n=4). Bars are split into the number of patients represented in each group.

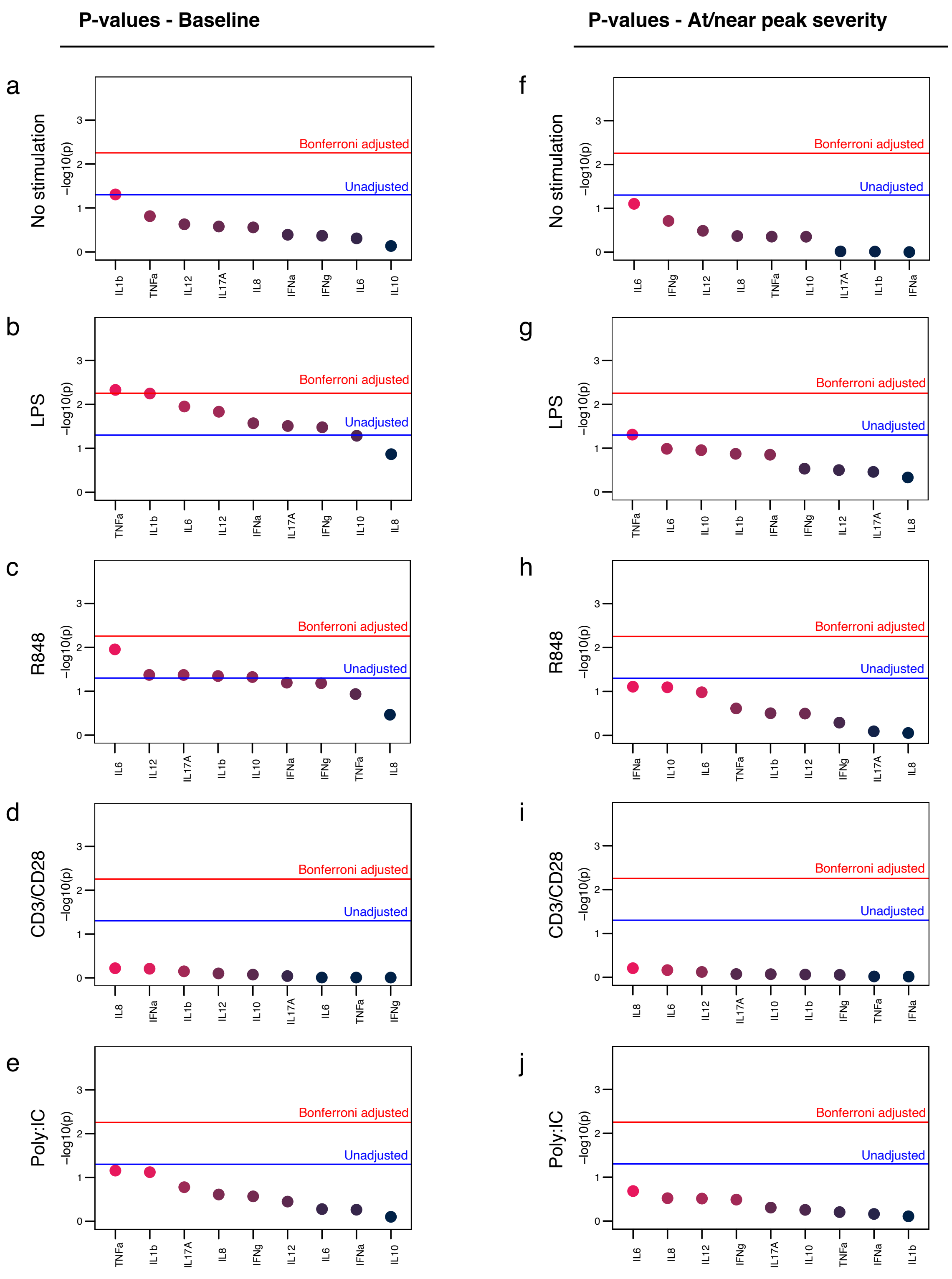


**Supplementary Figure 2. Significance of tested comparisons between severity groups at baseline and at/near peak severity .** **a-e,** p-values from Kruskal-Wallis tests comparing severity groups for each stimulus-response variable at baseline. **f-j,** p-values from Kruskal-Wallis tests comparing severity groups for each stimulus-response variable at/near peak severity. P-values are presented on a $-log_{10}$-axis. The threshold for statistical significance is shown before adjusting for multiple tests (p=0.05, blue line) and after Bonferroni-adjustment (9 tests: p=0.006, red line). Only p-values smaller than the Bonferroni corrected threshold were considered statistically significant. LPS, lipopolysaccharide; R848, resiquimod; CD, cluster of differentiation; Poly:IC, polyinosinic:polycytidylic acid; IFN, interferon; IL, interleukin; TNF, tumor necrosis factor; L O2, liters/minute of oxygen supply; ICU, intensive care unit.

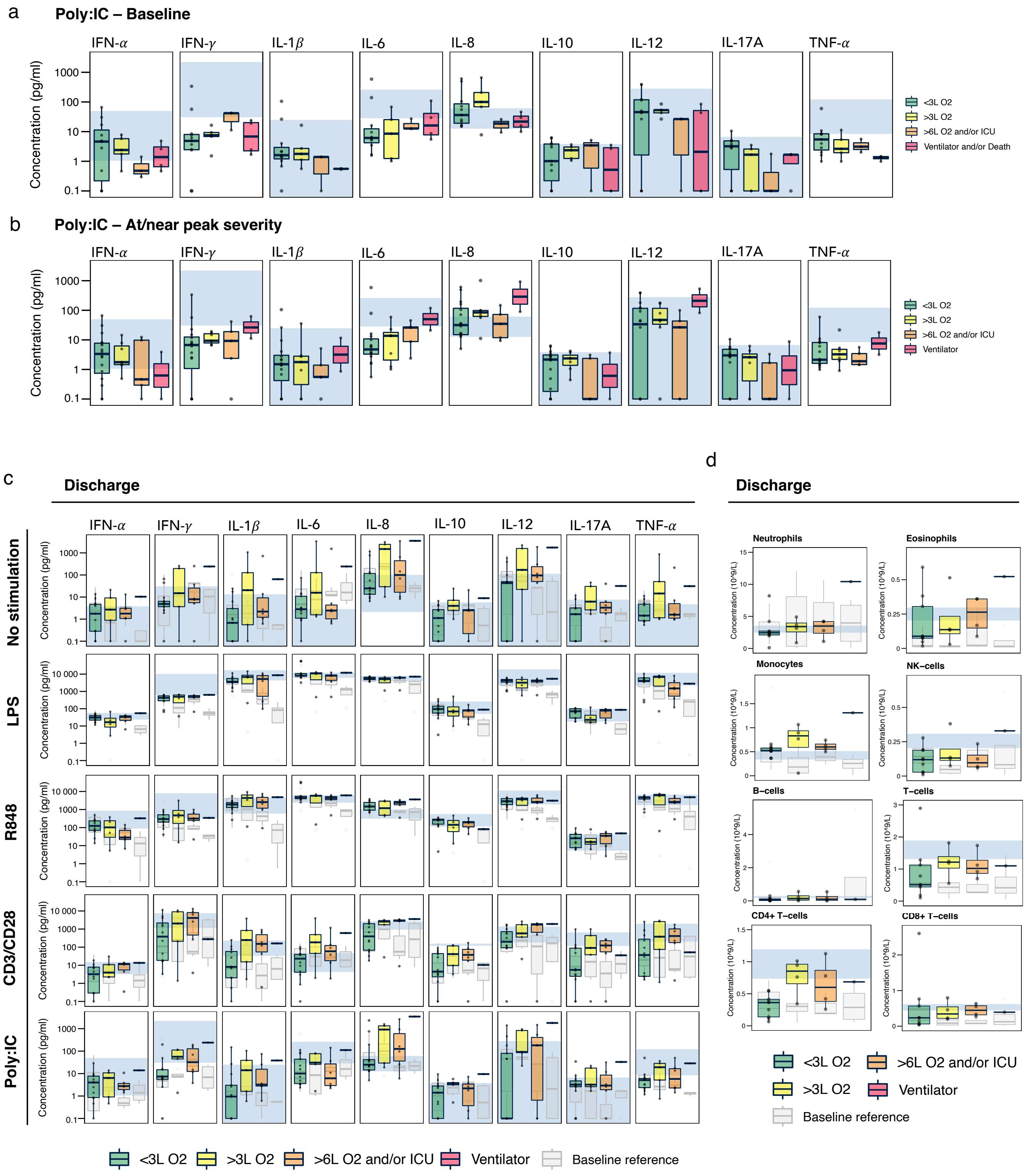


**Supplementary Figure 3. Poly:IC stimulated immune responses at baseline and at/near peak severity, and immune responses to all stimuli and immune cell constitution at discharge. a-b**, Cytokine levels in response to Poly:IC at (**a**) baseline (n=23), and (**b**) at/near peak severity (n=30). Patients are grouped based on peak severity grade at baseline (Grade 1 (n=11, green), Grade 2 (n=5, yellow), Grade 3 (n=3, orange), Grade 4 (n=4, red)) and grouped based on severity at time of sample collection at/near peak severity (Grade 1 (n=16, green), Grade 2 (n=7, yellow), Grade 3 (n=5, orange), Grade 4 (n=2, red)). **c,** Cytokine levels in response to no stimulation, LPS, R848, CD3/CD28, and Poly:IC at discharge, corresponding to the sample closest to discharge for each patient (n=25, except for CD3/CD28: n=24). Patients are grouped based on previous peak severity grade: Grade 1 (n=13, CD3/CD28: n=12, green), Grade 2 (n=5, yellow), Grade 3 (n=6, orange), Grade 4 (n=1, red). **d,** Immune cell subset counts at discharge (n=18). Patients are grouped based on previous peak severity: Grade 1 (n=9, green), Grade 2 (n=4, yellow), Grade 3 (n=4, orange), Grade 4 (n=1, red). Cytokine levels and immune cell subset counts are presented on a $log_{10}$ y-axis. Box edges represent the 25th and 75th percentiles, whiskers extend towards the most extreme values but no further than +/- 1.5 times the interquartile range from the hinge. Hollow dots beyond whiskers represent outliers. Solid dots represent individual patient measurements. Blue shaded areas represent the normal reference interval. Grey boxplots in background represent baseline levels. Poly:IC, polyinosinic:polycytidylic acid; IFN, interferon; IL, interleukin; TNF, tumor necrosis factor; L O2, liters/minute of oxygen supply; ICU, intensive care unit; LPS, lipopolysaccharide; R848, resiquimod; CD, cluster of differentiation; NK, natural killer.

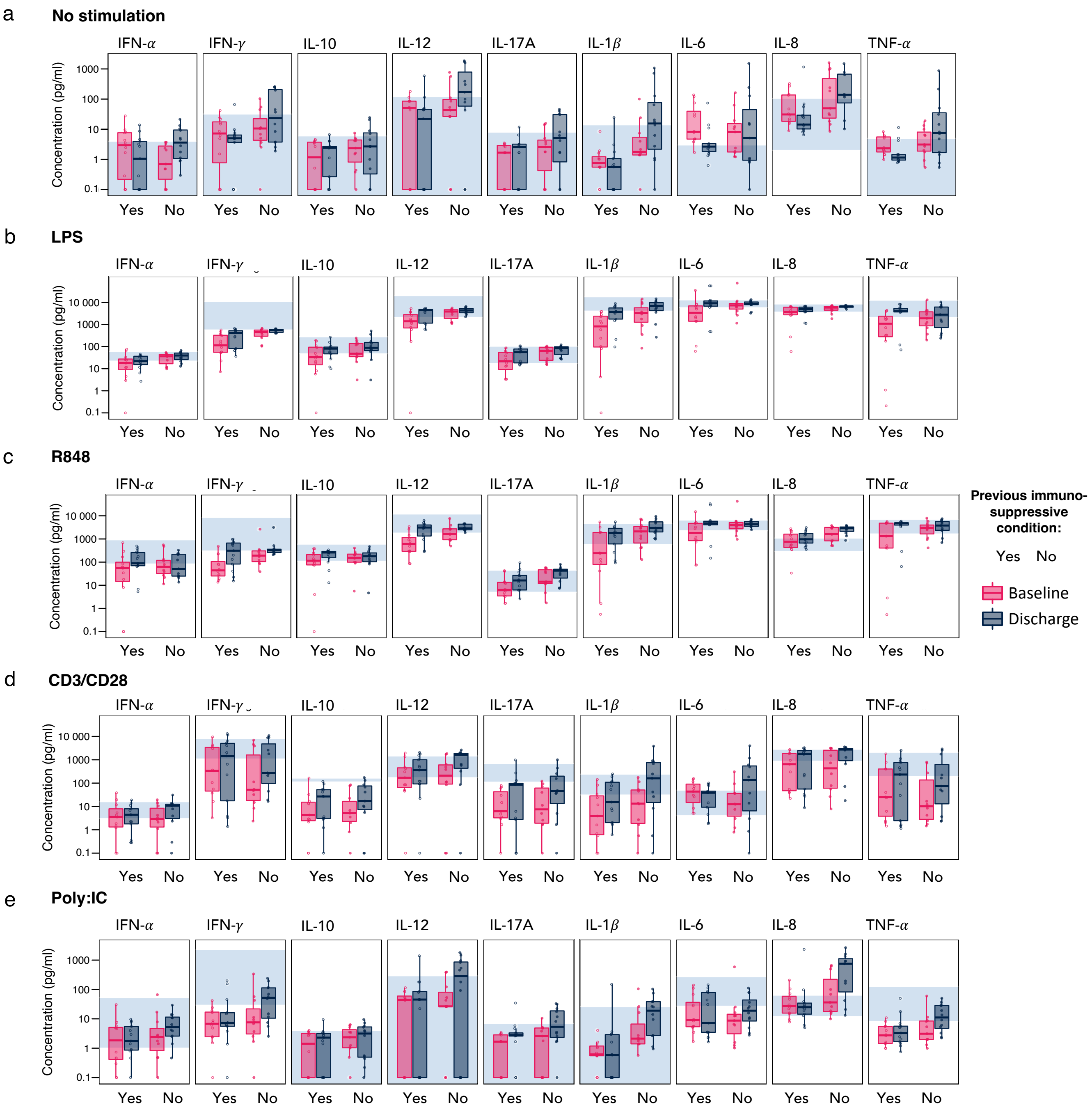


**Supplementary Figure 4. Stimulated immune responses at baseline vs discharge for patients with and without an immunosuppressive precondition. a-d**, Cytokine concentration levels in response to (**a**) no stimulation, (**b**) LPS (bacterial), (**c**) R848 (viral), (**d**) CD3/CD28 (T-cell receptor/co-receptor) and (**e**) Poly:IC, at baseline (n=23, pink boxes) vs discharge (n=25, blue boxes). Patients are grouped based on presence of an immunosuppressive precondition or not ("Yes": n=17,"No": n=13). Cytokine levels are presented on a $\log_{10}$ y-axis. Box edges represent the 25th and 75th percentiles, whiskers extend towards the most extreme values but no further than +/- 1.5 times the interquartile range from the hinge. Hollow dots beyond whiskers represent outliers. Solid dots represent individual patient measurements. Blue shaded areas represent the normal reference interval. LPS, lipopolysaccharide; R848, resiquimod; CD, cluster of differentiation; Poly:IC, polyinosinic:polycytidylic acid; IFN, interferon; IL, interleukin; TNF, tumor necrosis factor; L O2, liters/minute of oxygen supply; ICU, intensive care unit.

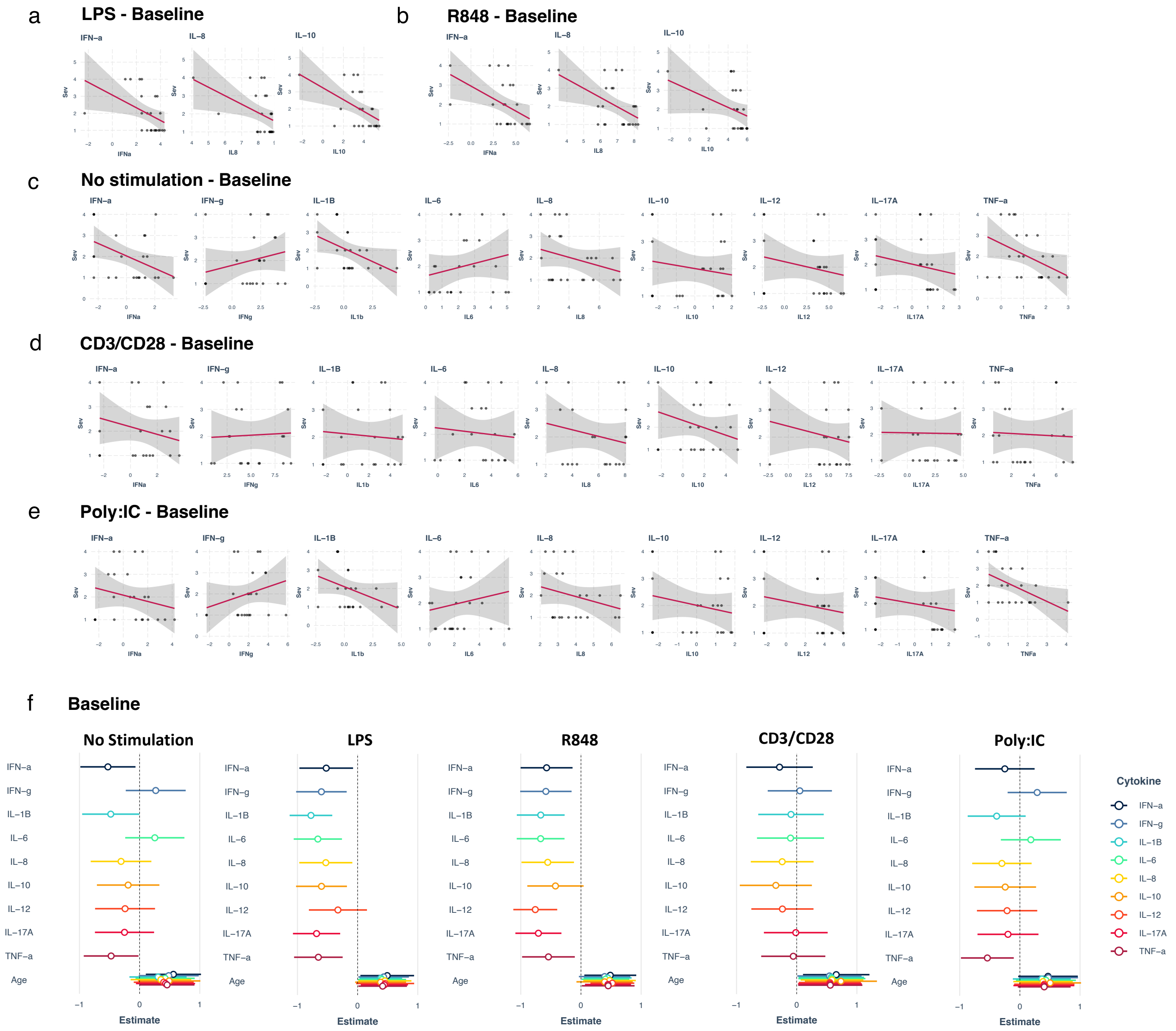


**Supplementary Figure 5. Associations between individual cytokine stimulus-response variables and future peak severity at baseline. a-e**, Associations after adjusting for age between individual cytokine variables (log-transformed cytokine concentrations) at baseline and future peak severity grade (Sev) for (**a**) LPS stimulation, (**b**) R848 stimulation, (**c**) no stimulation, (**d**) CD3/CD28 stimulation, (**e**) Poly:IC stimulation. Shaded areas behind regression lines represent 95% confidence intervals. **f**, Collected regression coefficient estimates for the stimulated cytokine variables and age by all stimuli at baseline, hollow dots represent the estimates, bars represent 95% confidence intervals. LPS, lipopolysaccharide; R848, resiquimod; CD, cluster of differentiation; Poly:IC, polyinosinic:polycytidylic acid; IFN, interferon; IL, interleukin; TNF, tumor necrosis factor; L O2, liters/minute of oxygen supply; ICU, intensive care unit.

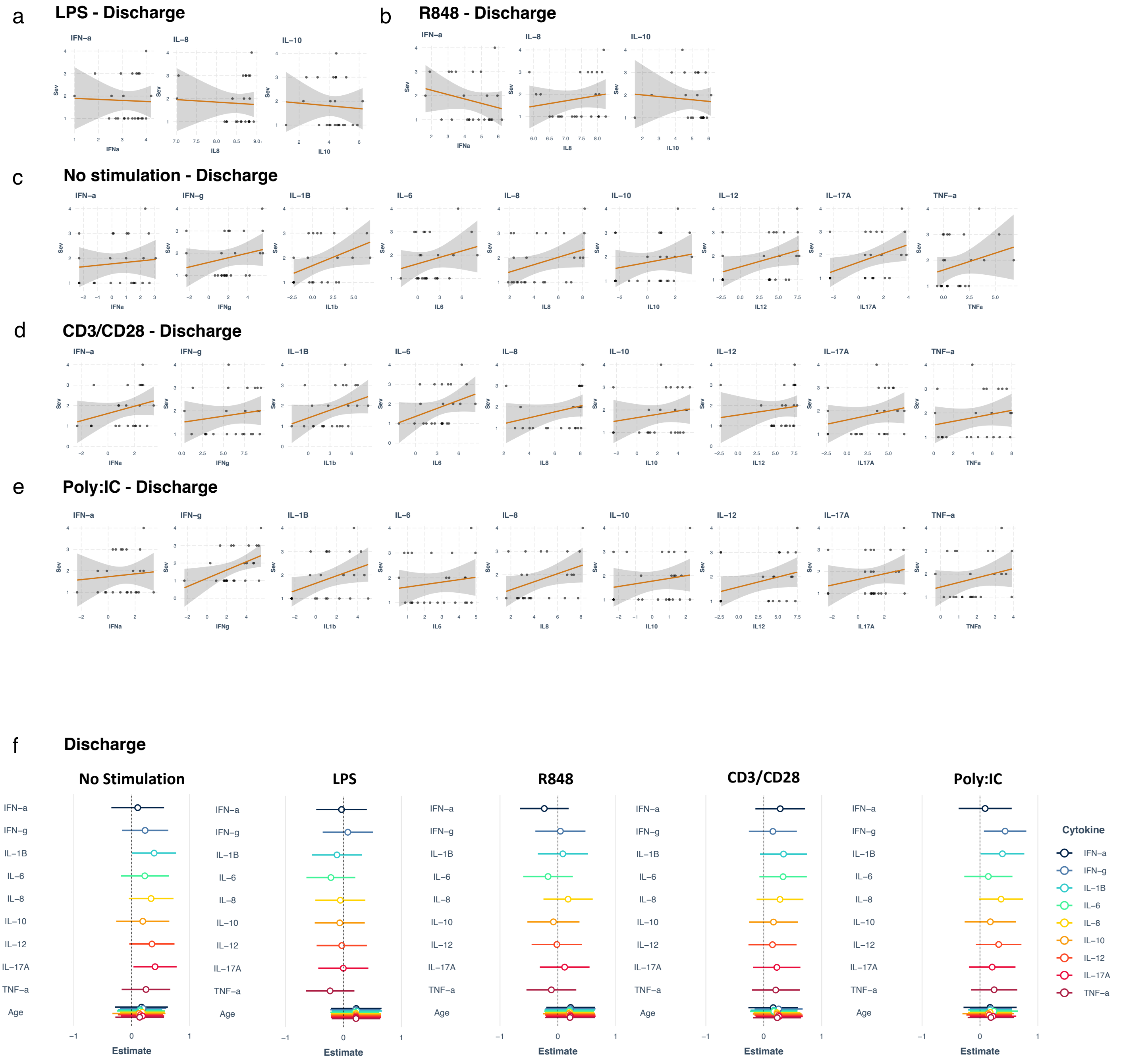


**Supplementary Figure 6. Associations between individual cytokine stimulus-response variables and previous peak severity at discharge. a-e**, Associations after adjusting for age between individual cytokine variables (log-transformed cytokine concentrations) at discharge and future peak severity grade (Sev) for (**a**) LPS stimulation, (**b**) R848 stimulation, (**c**) no stimulation, (**d**) CD3/CD28 stimulation, (**e**) Poly:IC stimulation. Shaded areas behind regression lines represent 95% confidence intervals. **f**, Collected regression coefficient estimates for the stimulated cytokine variables and age by all stimuli at discharge, hollow dots represent the estimates, bars represent 95% confidence intervals. LPS, lipopolysaccharide; R848, resiquimod; CD, cluster of differentiation; Poly:IC, polyinosinic:polycytidylic acid; IFN, interferon; IL, interleukin; TNF, tumor necrosis factor; L O2, liters/minute of oxygen supply; ICU, intensive care unit.

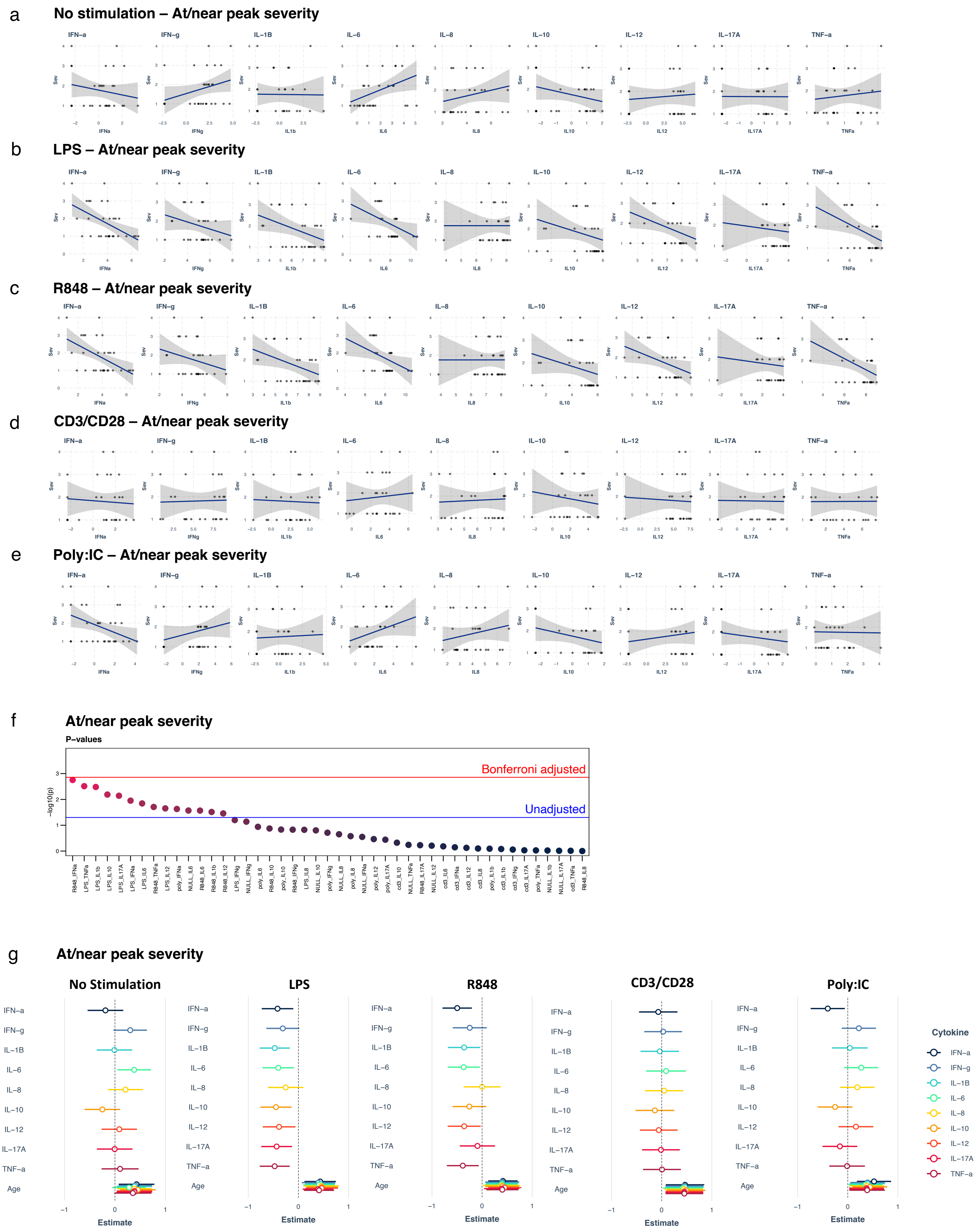


Supplementary Figure 7. Associations between individual cytokine stimulus-response variables and current severity at/near peak severity. **a-e**, Associations after adjusting for age between individual cytokine variables (log-transformed cytokine concentration, log(concentration)) at/near peak severity and current severity grade (Peak Severity) for (**a**) LPS stimulation, (**b**) R848 stimulation, (**c**) no stimulation, (**d**) CD3/CD28 stimulation, (**e**) Poly:IC stimulation. Shaded areas behind regression lines represent 95% confidence intervals. **f**, p-values from all linear regression analyses (n=45) after adjusting for age on a $-\log_{10}$-axis at/near peak severity. The threshold for statistical significance is shown before adjusting for multiple tests (p=0.05, blue line) and after Bonferroni-adjustment (45 tests: p=0.001, red line). Only associations with p-values smaller than the Bonferroni corrected threshold (p<0.001) were considered statistically significant. **g**, Collected regression coefficient estimates for the stimulated cytokine variables and age by all stimuli at/near peak severity, hollow dots represent the estimates, bars represent 95% confidence intervals. LPS, lipopolysaccharide; R848, resiquimod; CD, cluster of differentiation; Poly:IC, polyinosinic:polycytidylic acid; IFN, interferon; IL, interleukin; TNF, tumor necrosis factor, L O2, liters/minute of oxygen supply; ICU, intensive care unit.

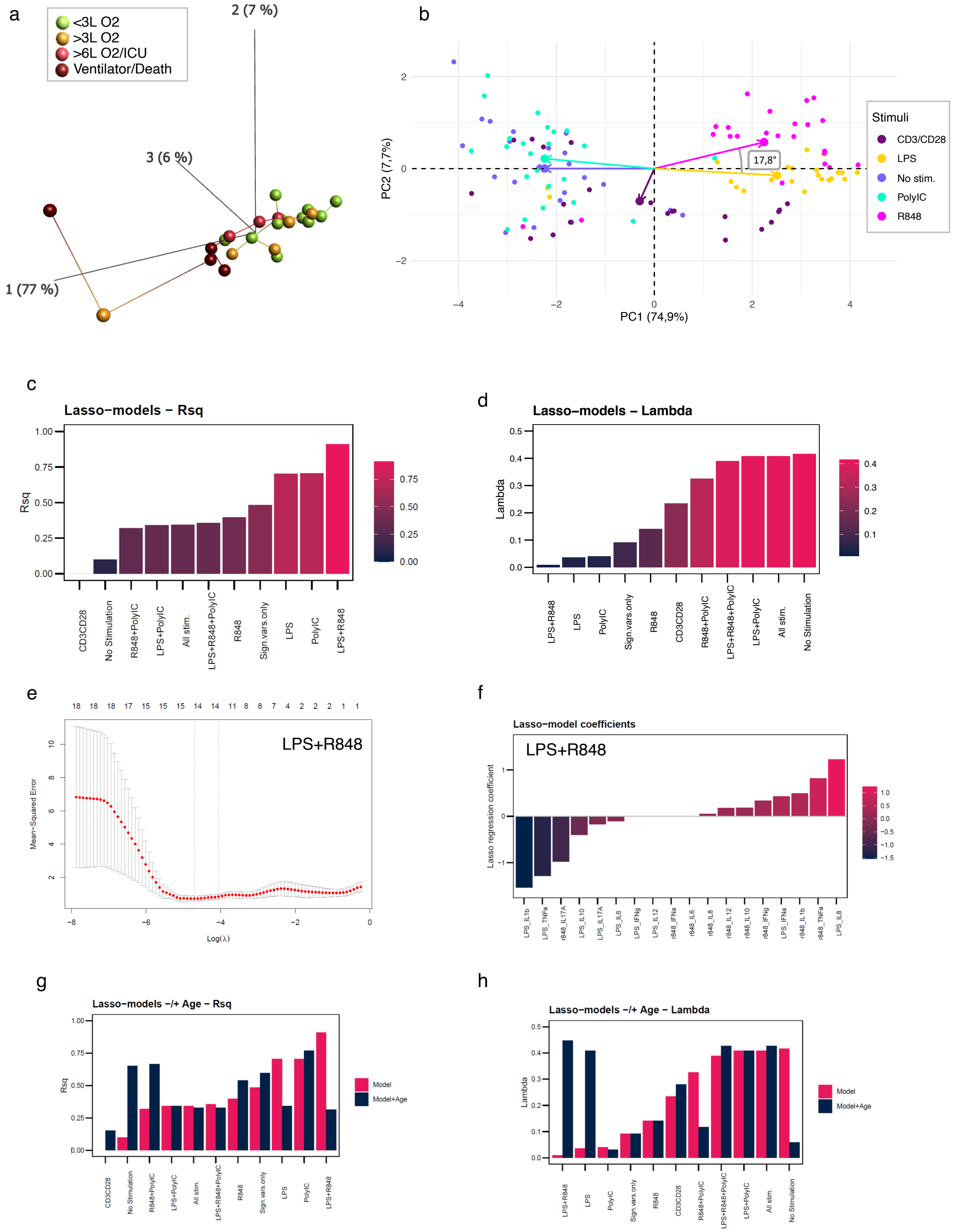

**Supplementary Figure 8. Principal component analyses of TruCulture data at baseline, and ordinary least squares in combination with LASSO penalties to identify a baseline signature for predicting peak severity. a**, Principal component analysis displaying severity levels based on LPS+R848 TruCulture baseline data. **b**, Principal component analysis displaying TruCulture data from all five stimuli at baseline. Arrows extend from origin to centroid of each stimulus representing the loadings of each stimuli in the projected dimensions. **c**, $R^2$ of the best model from each bin tested. **d**, Best lambda from each bin tested. **e**, The cross validation process for identifying the best lambda for the model based on combined LPS+R848 baseline data. **f**, The coefficients for the variables in the LPS+R848 model. Coefficients = 0 were considered insignificant for the model and thus excluded, all other coefficients were included. **g**, $R^2$ from all bins tested with and without including "Age" as a variable; red bars represent models excluding "Age", blue bars represent models including "Age". **h**, Best lambda from all bins tested with and without including "Age" as a variable; red bars represent models excluding "Age", blue bars represent models including "Age". L O2, liters/minute of oxygen supply; ICU, intensive care unit, PC, principal component; LPS, lipopolysaccharide; R848, resiquimod; CD, cluster of differentiation; Poly:IC, polyinosinic:polycytidylic acid; No Stim, no stimulation; All stim, all stimuli; Sign.vars.only, significant variables only (LPS_IL1B, R848_IL12 and R848_IL17A).

a

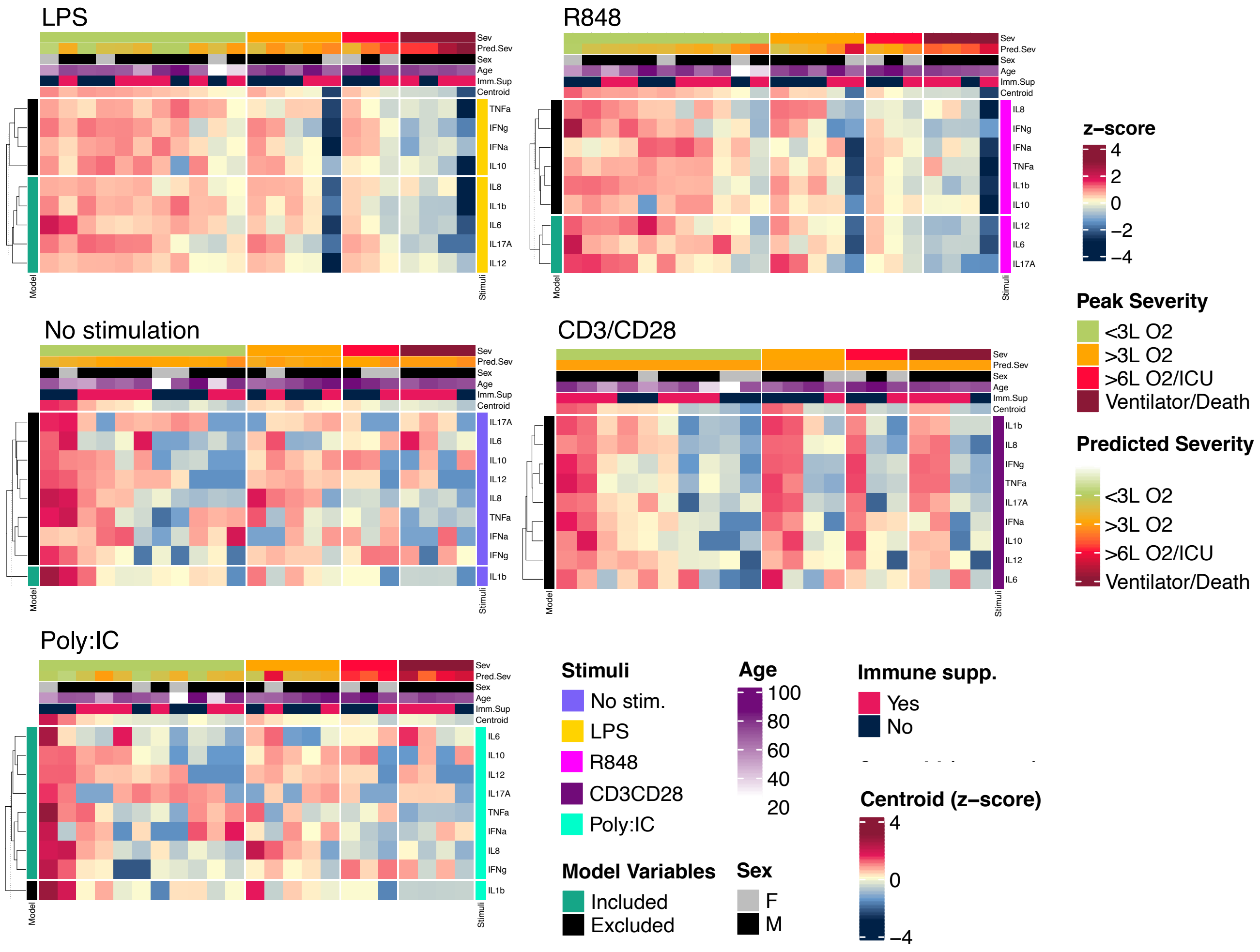


**Supplementary Figure 9. Stimulated immune responses by all stimuli at baseline. a**, Cytokine variables by each stimulus separately at baseline. Each column represents a patient, each row represents a stimulus-cytokine variable. Columns are grouped by future peak severity, rows are grouped by inclusion/exclusion in the corresponding LASSO regression model. Hierarchical clustering by Euclidean distance as dissimilarity metric was preformed within groups/splits (dendrogram only shown for rows). The top annotations represent (up-down): “Sev”: future peak severity, “Pred.Sev”: predicted severity in current cohort based on the LPS+R848 LASSO regression model, “Sex”: sex at birth, “Age”: age at time of inclusion in study, “Imm.Sup”: whether an immune-suppressive pre-condition was present, “Centroid”: the row mean value. Row annotations represent (left-right): “Model”: Inclusion/exclusion of variable in the LPS+R848 model, “Stimuli”: the stimulus for each cytokine variable. Data used for visualization were log-transformed and standardized. LPS, lipopolysaccharide; R848, resiquimod; CD, cluster of differentiation; Poly:IC, polyinosinic:polycytidylic acid; L O2, liters/minute of oxygen supply; ICU, intensive care unit.

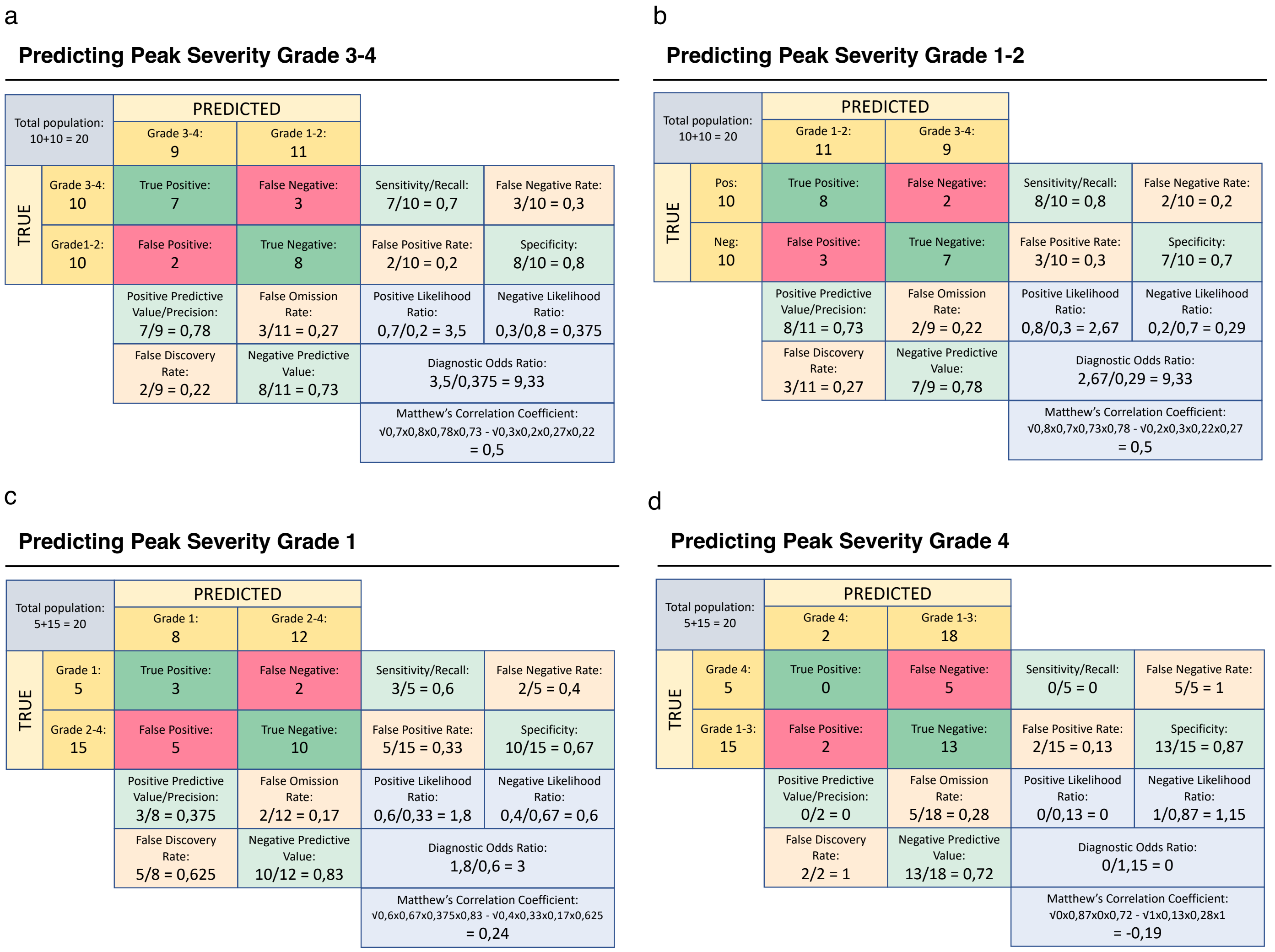


**Supplementary Figure 10. Validation of the immune response signature in a separate cohort.** Predictions were performed using the LPS+R848 model based on baseline TruCulture cytokine concentration data (standardized and log-transformed) from a separate validation cohort of hospitalized COVID-19 patients (n=20, 5 in each peak severity group). **a-d**, Calculations of sensitivity/recall, specificity, false positive rate, false negative rate, positive predictive value/precision, negative predictive value, false discovery rate, false omission rate, positive likelihood ratio, negative likelihood ratio, diagnostic odds ratio, and Mathew's correlation coefficient are presented for predicting (**a**) severity grade 3-4 vs 1-2, (**b**) severity grade 1-2 vs 3-4, (**c**) severity grade 1 vs 2-4, and (**d**) severity grade 4 vs 1-3.
