# Supplementary material for: Early stimulated immune responses predict clinical disease severity in hospitalized COVID-19 patients
Source: Commun Med (Lond). 2022 Sep 12;2:114. doi: 10.1038/s43856-022-00178-5 (PMC9466310; doi:10.1038/s43856-022-00178-5)
Supplement: Supplementary file 4 — Description of Additional Supplementary Files [file 43856_2022_178_MOESM4_ESM.pdf]

Description of Additional Supplementary Files

File name: Supplementary Data 1
Description: Source data for graphs presented in the main figures.

File name: Supplementary Data 2
Description: Source data for graphs presented in supplementary figures.
