# Supplementary material for: Early stimulated immune responses predict clinical disease severity in hospitalized COVID-19 patients
Source: Commun Med (Lond). 2022 Sep 12;2:114. doi: 10.1038/s43856-022-00178-5 (PMC9466310; doi:10.1038/s43856-022-00178-5)
Supplement: Supplementary file 6 — Reporting Summary [file 43856_2022_178_MOESM6_ESM.pdf]

nature portfolio

Corresponding author(s): Sisse Rye Ostrowski

Last updated by author(s): Aug 26, 2022

Reporting Summary

Nature Portfolio wishes to improve the reproducibility of the work that we publish. This form provides structure for consistency and transparency in reporting. For further information on Nature Portfolio policies, see our Editorial Policies and the Editorial Policy Checklist.

Statistics

For all statistical analyses, confirm that the following items are present in the figure legend, table legend, main text, or Methods section.

n/a	Confirmed	
☐	☒	The exact sample size (n) for each experimental group/condition, given as a discrete number and unit of measurement
☐	☒	A statement on whether measurements were taken from distinct samples or whether the same sample was measured repeatedly
☐	☒	The statistical test(s) used AND whether they are one- or two-sided<br>*Only common tests should be described solely by name; describe more complex techniques in the Methods section.*
☐	☒	A description of all covariates tested
☐	☒	A description of any assumptions or corrections, such as tests of normality and adjustment for multiple comparisons
☐	☒	A full description of the statistical parameters including central tendency (e.g. means) or other basic estimates (e.g. regression coefficient) AND variation (e.g. standard deviation) or associated estimates of uncertainty (e.g. confidence intervals)
☐	☒	For null hypothesis testing, the test statistic (e.g. F, t, r) with confidence intervals, effect sizes, degrees of freedom and P value noted<br>*Give P values as exact values whenever suitable.*
☒	☐	For Bayesian analysis, information on the choice of priors and Markov chain Monte Carlo settings
☒	☐	For hierarchical and complex designs, identification of the appropriate level for tests and full reporting of outcomes
☐	☒	Estimates of effect sizes (e.g. Cohen's d, Pearson's r), indicating how they were calculated

Our web collection on statistics for biologists contains articles on many of the points above.

Software and code

Policy information about availability of computer code

Data collection	Sunhedsplatformen electronic health records (EHR), Kaluza Analysis 2.1
Data analysis	R software version 4.0.3, packages "ggplot", "stats", "corrplot", "ComplexHeatmap", "glmnet", "jtools", "networkD 2" Qlucore Omics Explorer

For manuscripts utilizing custom algorithms or software that are central to the research but not yet described in published literature, software must be made available to editors and reviewers. We strongly encourage code deposition in a community repository (e.g. GitHub). See the Nature Portfolio guidelines for submitting code & software for further information.

Data

Policy information about availability of data

All manuscripts must include a data availability statement. This statement should provide the following information, where applicable:

- Accession codes, unique identifiers, or web links for publicly available datasets
- A description of any restrictions on data availability
- For clinical datasets or third party data, please ensure that the statement adheres to our policy

This work was based on a small cohort of subjects who are part of the bigger COVIMUN study. Raw data cannot yet be made publicly available due to data privacy issues according to EU legislation. However, anonymized source data for each figure is provided in Supplementary Data 1 and 2. Raw data will be made available in accordance with EU regulations for data privacy once these have been published – hopefully within 12 months, please contact the corresponding author regarding requests.

Field-specific reporting

Please select the one below that is the best fit for your research. If you are not sure, read the appropriate sections before making your selection.

☒ Life sciences ☐ Behavioural & social sciences ☐ Ecological, evolutionary & environmental sciences

For a reference copy of the document with all sections, see nature.com/documents/nr-reporting-summary-flat.pdf

Life sciences study design

All studies must disclose on these points even when the disclosure is negative.

Sample size	This paper inlcuded data on the first 30 patients in the COVIMUN study which has since incuded more patients and thus constituetes a much larger cohort. We debated that 30 patients was sufficient for the work presented in the current paper, where the primary purpose was descriptive analysis and hypothesis generation. In the revised manuscript we selected an additional 20 patients from the COVIMUN study for validation of our findings. Moving forward, we aim at furhter exploring and validating the findings and hypotheses generated based on the current work in the full COVIMUN cohort.
Data exclusions	All criteria for industion/exdusion of data were defined prior to data analysis. Data were exduded if a sample was taken outside of the defined timeframe for the timepoints defined in this study (Baseline, at/near Peak Severity, Discharge). This specifically affected the baseline samples, which had to be collected within 7 days of hospitalization, and doser to hospital admission than to discharge. Thus, 7 patients did not have a sample collected that matched the baseline criteria. Similarly, for discharge samples from patients with short ad missions, the sample needed to be taken doser to discharge than to ad mission, why some samples were excluded for this reason. Otherwise no data was excluded, however samples could be missing due to 1) the flow cytometry ana lyses were not run in weekends, therefore samples for flow cytometry were sometimes missing, 2) in the TruCulture analysis, the CD3/CD28 stimulus was implemented after the inclusion of the first three subjects, therefore measurements from this stimulus are missing for these patients at the first timepoints, 3) patients dying (n=2) or 3) patients being transferred to another hospital du ring the study (n=l). For all patients who had samples missing that corresponded with any of the defined time-points, full mapping of clinical disease trajectories was still completed.
Replication	No replication has been done in this study as it is a longitudinal study with human individuals.
Randomization	There was no randomization in this study. As we aimed to investigate immunological changes in patients with different disease trajectories, patients were manually grouped into four groups based on four pre-defined grades of disease severity du ring hospitalization. The grading system and grouping was decided on prior to any data visualization or data ana lyses. Grade 1 was defined as requiring less than 3 liters per min ute (L/min) of supplemental oxygen (02) to keep peripheral blood oxygen saturation (SAT) > 92%; Grade 2 was defined as requiring more or equal to 3 but <6 L/min of 02 to maintain SAT > 92%; Grade 3 was defined as requiring more or equal to 6 L/min of 02 and/or being admitted to the ICU; and Grade 4 was defined as being treated with mechanical ventilator support. Patients who died during hospitalization were grouped together with patients with peak severity Grade 4 in the analyses.
Blinding	The aim of this presented work was unknown to the lab technicians who collected and analysed patient blood samples, as all practical execution of the analyses presented in this study are part of rutine clinical and/or laboratory analyses at our hospital.

Reporting for specific materials, systems and methods

We require information from authors about some types of materials, experimental systems and methods used in many studies. Here, indicate whether each material, system or method listed is relevant to your study. If you are not sure if a list item applies to your research, read the appropriate section before selecting a response.

Materials & experimental systems

n/a	Involved in the study	
☐	☒	Antibodies
☒	☐	Eukaryotic cell lines
☒	☐	Palaeontology and archaeology
☒	☐	Animals and other organisms
☐	☒	Human research participants
☒	☐	Clinical data
☒	☐	Dual use research of concern

Methods

n/a	Involved in the study	
☒	☐	ChIP-seq
☐	☒	Flow cytometry
☒	☐	MRI-based neuroimaging

Antibodies

Antibodies used	The antibodies used were alle from Beckman Coulter: CD61-FITC Clone SZ21, CD69-PE Clone TPl.55.3, CD16-ECD Clone 3G8, CD56-PCS.5 Clone N901, CD19-PC7 Clone J3-119, CD8-APC Clone B9.11, CD4-APC-A700 Clone 13B8.2, CD3-APC-A750 Clone UCHTl, CD14-PBE Clone RMO52 and CD45-KrO Clone J33.

Validation	All antibodies used were in a single tube with a premixed cocktail of dry antibodies. The tube was tested and validated by the manufacturer and validated in-house afterwards.

Human research participants

Policy information about studies involving human research participants

Population characteristics	In the original patient cohort the median age was 70 years (range 26-89), and two thirds (67%) of the patients were male. The median body mass index was 24.5 kg/m2 (range 15-43). 57% of patients had pre-existing conditions associated with immunosuppression prior to hospitalization. Such conditions included severe multimorbidity, acquired immunodeficiency syndrome (AIDS), active malignancy with recent chemotherapy treatment, or ongoing immunosuppressive treatment. 87% of patients had at least one comorbidity, 37% of patients had at least two comorbidities. All patient characteristics are summarized in Extended Data Supplementary Tables 1 and 2.<br>In the validation cohort the median age was 69 years, 70% were male, and 30% of patients had pre-existing conditions associated immunosuppression.
Recruitment	Patients were identified and recruited upon hospitalization due to COVID-19. Patients were included based on the criteria 1) PCR-confirmed SARS-CoV-2 infection, 2) hospitalization due to COVID-19, and 3) informed consent given to the study. Since the study began recruitment at Rigshospitalet (the main high-specialty hospital in the region) the majority of patients were recruited at Rigshospitalet, and therefore proportion of patients with conditions associated with immune dysfunction may be higher in our cohort than in the general population.
Ethics oversight	This study was approved by the Ethical Committee (H-20026502) and Data Protection Agency (P-2020-426).

Note that full information on the approval of the study protocol must also be provided in the manuscript.

Flow Cytometry

Plots

Confirm that:

- [ ] The axis labels state the marker and fluorochrome used (e.g. CD4-FITC).
- [ ] The axis scales are clearly visible. Include numbers along axes only for bottom left plot of group (a 'group' is an analysis of identical markers).
- [ ] All plots are contour plots with outliers or pseudocolor plots.
- [x] A numerical value for number of cells or percentage (with statistics) is provided.

Methodology

Sample preparation	Peripheral blood was sampled in sodium-citrate K2-EDTA tubes and transferred immediately to the laboratory. Analyses were performed on fresh whole-blood within 24 hours of collection.
Instrument	Navios Ex flowcytometer from Beckman Coulter
Software	Kaluza Analyses 2.1 from Beckman Coulter
Cell population abundance	Cell populations were reported in absolute concentrations. The tube contained a known number of beads (round 90.000) for calculation of absolute concentrations of the different cell populations. 100 ul blood were added to the tube and in the following flow analysis beads and cells were counted in parallel until 10.000 beads were reached. Absolute concentrations were calculated: [Concentration]=[cells counted] / (100ul x ([beads counted]/ [beads in tube])

Gating strategy

CD45/SS INT: Alive cells and beads are defined as CD45pos cells [CD45pos + beads].
[CD45pos + beads] CD61/CD14: discrimination between cells and beads [CD45pos] and [Beads].
[CD45pos] FS INT/FS PEAK: Singlets are defined (Area vs height) [Singlets].
[Singlets] FS INT/SS INT: Single cells without beads, overview.
[Singlets] CD45/SS INT: Granulocytes are defined as CD45low/SS high [Granulocytes]. Rest are definesd as non granulocytes [Non granulocytes].
[Granulocytes] CD16/SS INT: Neutrophil granulocytes are defined as SS high/CD16 pos [SSh_CD16pos]. Eosinophils are defined as SS high/CD16 neg [SSh_CD16neg].
[Non granulocytes] CD14/SS INT: Monocytes are defined as CD14pos/SS low-int [CD14pos] and T-, B, and NK-cells defined as CD14 neg/SS low [CD14neg/SS low].
[CD14neg/SS low] CD3/CD19: 'T-cells are defined as CD3pos/CD19neg [CD3pos], B-cells are defined as CD19pos/CD3neg [CD19pos] and NK-cells as CD3neg/CD19neg [Non T- and B-cells].
[CD3pos] CD4/CD8: CD4 pos T-cells are defined as CD4pos/CD8neg [CD4pos], CD8 pos T-cells are defined as CD8pos/CD4neg [CD8pos] and double negative T-cells as CD4neg/CD8neg [CD4negCD8neg].
[Non T- and B-cells] CD16/CD56: NK-cells are divided in tree populations; CD16pos/CD56pos, CD16pos/CD56neg and CD16neg/CD56pos.
[CD3pos] CD16/CD56: NKT-cells are defined as CD16neg/CD56pos.

[...] indicate a gated population

☐ Tick this box to confirm that a figure exemplifying the gating strategy is provided in the Supplementary Information.
